# Rapid Fishery Assessment by Market Survey (RFAMS) – An Improved Rapid-Assessment Approach to Characterising Fish Landings in Developing Countries

**DOI:** 10.1371/journal.pone.0109182

**Published:** 2014-10-02

**Authors:** William T. White, Peter R. Last, Dharmadi, Ria Faizah, Umi Chodrijah, Rik C. Buckworth, Catherine M. Dichmont

**Affiliations:** 1 CSIRO Oceans & Atmosphere Flagship, Hobart, Tasmania, Australia; 2 Research Centre for Fisheries Management and Conservation, Agency for Marine and Fisheries Research and Development, Ministry of Marine Affairs and Fisheries, Jakarta, Indonesia; 3 CSIRO Oceans & Atmosphere Flagship, Dutton Park, Queensland, Australia; Aristotle University of Thessaloniki, Greece

## Abstract

The complex multi-gear, multi-species tropical fisheries in developing countries are poorly understood and characterising the landings from these fisheries is often impossible using conventional approaches. A rapid assessment method for characterising landings at fish markets, using an index of abundance and estimated weight within taxonomic groups, is described. This approach was developed for contexts where there are no detailed data collection protocols, and where consistent data collection across a wide range of fisheries types and geographic areas is required, regardless of the size of the site and scale of the landings. This methodology, which was demonstrated at seven fish landing sites/fish markets in southern Indonesia between July 2008 and January 2011, provides a rapid assessment of the abundance and diversity in the wild catch over a wide variety of taxonomic groups. The approach has wider application for species-rich fisheries in developing countries where there is an urgent need for better data collection protocols, monitoring future changes in market demographics, and evaluating health of fisheries.

## Introduction

Indonesian fisheries are among the largest and most productive worldwide, and are critical to the nation's economic development and in providing food resources to millions of people. In 2004, production from marine capture fisheries in Indonesia was approximated at 4.5 million tonnes with an estimated gross value of US $3.13 million [1]. The total marine capture fisheries production in Indonesia increased to 5.4 million tonnes in 2010 [2]. The high level of employment, which in 2003 was estimated at 3.3 million people directly employed in marine capture fisheries [1], is an important indicator of the value of fisheries to Indonesia. This places Indonesian marine capture fisheries among the top five worldwide in terms of fisheries production. The level of fishing effort in Indonesia is also increasing rapidly, with the number of motorised marine vessels increasing from an estimated 348,425 in 2007 to 390,770 in 2009 [3].

The majority of Indonesia's capture fisheries are considered to be fully or overexploited despite the fact that marine capture production in Indonesia is increasing annually [4]. One of the greatest problems facing Indonesia's Ministry of Marine Affairs and Fisheries is the quality of fishery statistics available. Since Indonesia's fisheries are both multi-gear and multi-species fisheries operating over 80,000 km of coastline, it would not be feasible to collected catch statistics from all catches. As a result, catch statistics are obtained by averaging daily catch from a small subset of vessels and multiplying by the number of vessels and number of fishing days to obtain annual catches [5]. This method has many obvious weaknesses, with a major problem being that illegal, unregulated and unreported (IUU) catches are not accounted for in these statistics [3], [6], [7].

Catches of many small-scale fisheries are often overlooked, but the cumulative catches from this sector can be very high, particularly in biologically rich and densely populated countries such as Indonesia. In developing countries these small scale-fisheries are also extremely important to the local economy and account for a substantial proportion of the overall capture production [8], [9]. Despite this, there are very few studies of multi-gear, multi-species fisheries in tropical regions of the world [10], [11], [12], hindering the ability to develop adequate management strategies for such areas. [12] undertook comprehensive surveys of the main fish market at Manado, North Sulawesi (Indonesia), which was dominated by small-scale coral reef fisheries. These market surveys recorded species-level composition data where possible and allocated fishes into 10 cm size-classes. This approach allows for an accurate assessment of the landings at this particular market, but obtaining this level of information at much larger and demographically more complex markets would not be possible. There is a need for a more rapid, standardised method of collecting landings information from fish markets or landing sites in tropical regions with diverse fisheries.

In this paper, we present a new Rapid Assessment Protocol: Rapid Fisheries Assessment by Market Survey (RFAMS) for assessing catch composition at fish landing sites and markets in the circumstances where no detailed data collection protocols exist. Present methods of recording catch composition data in developed and small-scale fisheries are not suitable for countries such as Indonesia which have very large, complex, multi-gear and multi-species fisheries. We emphasise the importance of collecting accurate taxonomic information, albeit not to species level, to enable biodiversity to be assessed. The major aim of this new data collection methodology is to provide data-poor regions with a tool for being able to efficiently characterise the landings at different fish landing sites and markets regardless of their physical size and the scale of the landings. This method is considered to be widely applicable to fisheries researchers and managers in data-poor regions where there is no existing, or minimal formalised, market data collection protocols. The data collected using this approach will also provide important benchmarks for monitoring future changes in these fisheries.

## Methods

### 1. Ethics Statement

All marine life examined in this study were landed from small-scale fisheries in Indonesia and were already dead upon inspection. Permission to undertake surveys in Indonesia was granted by the Research Centre for Fisheries Management and Conservation in Jakarta as part of a collaborative project funded by the Australian Centre for International Agricultural Research (project code FIS2006\142).

### 2. Study Area

**Figure 1:**
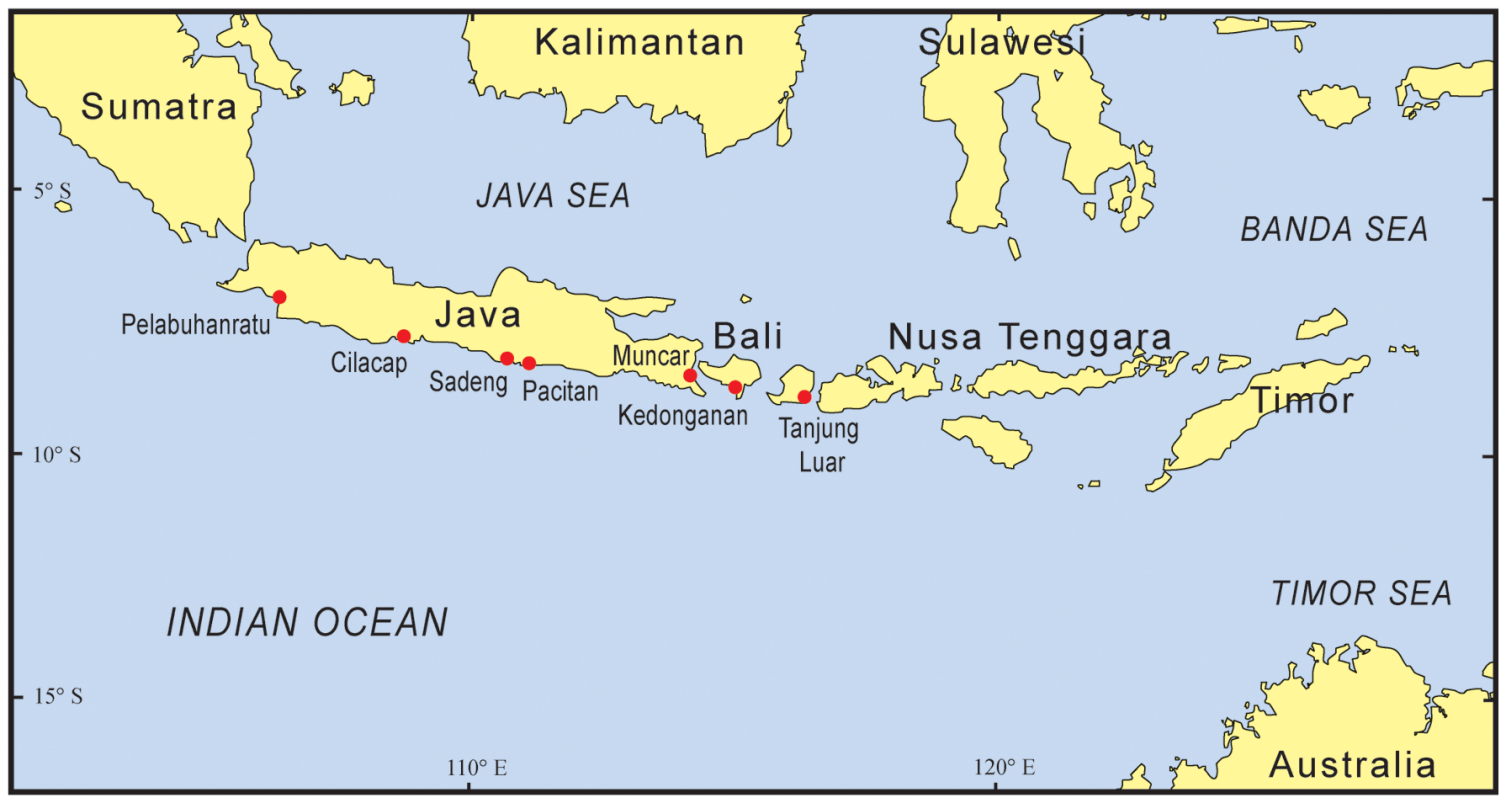
Map of eastern Indonesia. Fish landing sites surveyed in this study.

Seven fish landing sites were surveyed across five provinces in southern Indonesia, i.e. West Java (Pelabuhanratu), Central Java (Cilacap and Sadeng), East Java (Muncar and Pacitan), Bali (Kedonganan) and West Nusa Tenggara (Tanjung Luar in Lombok) (Fig. 1). Three of these sites, Muncar, Pacitan and Sadeng, were surveyed 3 or 4 times, while the remaining sites were surveyed between 6 and 9 times during the study. Across these 7 sites, a total of 60 daily surveys were completed between July 2008 and January 2011.

Pelabuhanratu is a medium-sized port on the south coast of West Java with a diverse range of fisheries operating, from *bagans* (light traps) to oceanic longlining. Cilacap is a large port on the south coast of Central Java which is dominated by a tuna longline fishery. Sadeng is a small port on the south coast of Central Java, east of Cilacap, with only small landings from several different fisheries. Pacitan is a small port on the southwest coast of East Java which is dominated by a trolling fishery targeting small tuna. Muncar is a large port on the east coast of East Java which is dominated by a purse-seine fishery for lemuru (sardines). Kedonganan is a medium to small landing site at Jimbaran Bay in southern Bali which has a diverse range of fisheries targeting a variety of fish and invertebrate groups. Tanjung Luar is a medium-sized landing site on the east coast of Lombok which is best known for its longline fishery targeting large sharks and large mobulid bycatch from the tuna gillnet fisheries.

### 3. The Rapid Fisheries Assessment by Market Survey (RFAMS) methodology

The data sheets used for RFAMS can be found in Datasheet S1. The initial information recorded is the fish market (or landing site/port), data recorder (person or group), date and time of survey (e.g. 7:00–9:00 am). The total estimated weight of the landings was classified into one of five categories, i.e. <100 kg, 100–500 kg, 500 kg to 1 tonne, 1–10 tonnes and>10 tonnes. Where possible, a total estimated weight was also provided calculated by summing the estimated weights in each of the taxonomic categories (see below). Where possible, the fleet size was recorded by determining the approximate number of boats in each of six categories based on boat size (note GT = gross tonnes): <5 GT, 5–10 GT, 10–30 GT,>30 GT, small with outboards and small without outboards.

To capture data on the broad habitats being fished, the estimated percentage contribution of the catches in each predetermined bathome (depth-related habitat zone) was also recorded. Generalised bathomes were used in this study: 1) freshwater; 2) estuarine; 3) coastal; 4) coral reef; 5) shelf demersal; 6) slope demersal; 7) inshore pelagic; 8) oceanic; 9) aquaculture; 10) intertidal. Likewise, to capture data on the various fisheries involved in the landings, the estimated percentage contribution of the catches in each predetermined fishing method was also recorded. The predetermined fishing methods used for Indonesia in this study were: 1) cast net; 2) diver/spear; 3) dredge; 4) gillnet; 5) handline; 6) longline; 7) traps; 8) purse seine; 9) tidal trap; 10) trawl; 11) beach seine; 12) *bagan* (light trap); 13) by hand; 14) trammel net; 15) hand net; 16) dynamite/poisoning.

Catch composition of the landings was divided into 6 major taxonomic groupings: Cephalopods (e.g. squid, octopus), Other Molluscs (e.g. bivalves, clams), Crustaceans (e.g. prawns, crayfish and crabs), Other (e.g. marine mammals, sea turtles, algae), Elasmobranchs (e.g. sharks, rays) and Teleosts (bony fishes). For each of these major groupings, their percentage contribution to the landings (calculated from estimated weights of each of the taxonomic categories for all groups) and an estimate of the number of species observed was recorded.

Within each of the major taxonomic groupings, catch information was recorded on a family-level basis (e.g. Octopodidae, Penaeidae), except in a few cases where broader categories were used due to difficulties in accurate family-level identification during rapid surveys (e.g. squids, algae) (see Appendix S1). In the case of elasmobranchs, catch information was recorded at a species level due to the detailed knowledge available on this group from the Indonesian fish markets surveyed in this study [13]. In the case of the most abundant or most diverse teleost families, catch data was also recorded for the major genera or species groups, i.e. Carangidae (*Caranx/Carangoides*, *Decapterus*, *Scomberoides*, other carangids), Scombridae (*Thunnus/Euthynnus*, *Katsuwonus/Sarda*, *Scomberomorus/Acanthocybium*, other scombrids). The predetermined taxonomic categories provided in the data sheets (Datasheet S1) were based on surveys undertaken in Indonesia prior to this study (e.g. see [13]) as well as general knowledge of the fish groups exploited in South-east Asia. Blank spaces are also provided for other families encountered which are not listed.

Within each of the taxonomic categories, the data recorded were: an index of abundance, bathome, fishing method and estimated weight. An index of abundance (0–11) was recorded based on a log scale: 0 = 0; 1 = 1; 2 = 2–3; 3 = 4–9; 4 = 10–27; 5 = 28–81; 6 = 82–251; 7 = 250–749; 8 = 750–1999; 9 = 2000–5999; 10 = 6000–19,999; 11 = >20,000 individuals. A log scale index was used as it provides a good indication of abundance and is simple and quick to record which is important in fish markets where large numbers of some fish groups are landed. The corresponding number for the relevant bathome and fishing method for each taxonomic category was recorded (see above). For example, tuna caught by pelagic longlines would have a bathome of 8 (oceanic) and fishing method of 6 (longline). If there was multiple bathomes or fishing methods for a particular taxonomic category, only the major bathome and fishing gear was recorded. A total weight (kg) for each taxonomic category recorded was estimated. Estimation of weight was usually determined using known container weights that fish were stored in (e.g. Styrofoam boxes, cane baskets, plastic tubs and drums).

### 4. Multivariate analyses

The average estimated weight (kg) data for each family (or proxy taxonomic grouping, e.g. squids) at each of the 7 landing sites on each of the survey trips were used to construct a resemblance matrix, following fourth-root transformation, employing the Bray-Curtis similarity coefficient. The resulting matrix was then subjected to non-metric multidimensional scaling (nMDS) ordination and hierarchical cluster analysis using Primer v6 package [14]. A one-way Analysis of Similarities (ANOSIM) was employed to test whether the catch composition of the landings at each site were significantly different. Where significant differences exist, the magnitude of the R-statistic (0, no significant differences; 1, differences highly significant) was used to ascertain the extent to which the catch compositions of a priori groups differed. Multivariate Dispersion (MVDISP) was used to determine the degree of dispersion of each of the sites on ordination plots [15] and Similarity Percentages (SIMPER) was employed to determine the families that typified particular groups and/or contributed most to the dissimilarities between groups [16].

The same approach was also followed to analyse the catch composition by major taxonomic group (Cephalopods, Other molluscs, Crustaceans, Other, Elasmobranchs and Teleosts), by functional group (e.g. reef fish-herbivore, pelagic rays, inshore pelagics) and by a bathome/gear combination (e.g. coral reef/handline, shelf demersal/gillnet). The ‘functional group’ category was based roughly on the ecological niche that each family best fits, e.g. surgeonfishes (Acanthuridae) into reef fish-herbivore, devilrays and manta rays (Mobulidae) into pelagic rays (see Table S1 for full list). Any bathome/gear combination used which was recorded less than 10 times were put into a single category called ‘Rare’ (see Table S2 for full list).

## Results and Discussion

### 1. Understanding dynamics of the survey sites

In order to undertake RFAMS successfully, it was important to understand the basic dynamics of the fish landing site or market being surveyed. The most critical information is the timing and duration of landings. Preliminary information on the dynamics of the sites surveyed in this study provided key information on when to commence the survey and approximate daily duration of landings at each site. Landing sites and/or markets varied greatly not only in their catch composition but also in their dynamics. For example, Tanjung Luar is a moderately large landing site on the east coast of Lombok where catches are brought into the site directly from fishing boats or from neighbouring islands via trucks. This market commences at about 6:00 am, usually begins to slow around 8:00 am, and by 10:00 or 11:00 am is completely empty with no more activity. In contrast, the larger fishing port of Cilacap has landings commence around 8:00 am but continues off and on for most of the day, with some catches of prawns not being landed until 7:00 or 8:00 pm. Without accurate information on timing of landings at the sites being surveyed, large components of the catches can be completely missed.

The spatial dynamics at each site also need to be taken into account on each new survey trip to ensure no changes in landing sites have occurred. For example, at Cilacap, the area where catches from the trammel net fishery are landed changed multiple times between 2008 and 2011. Local fisheries officers were able to provide information where landings had been relocated. At this same location, the tuna landings from the tuna longline fishery, which often dominate landings at this site, was relocated to a different location during one survey trip due to dredging in the harbour. Without the local knowledge of these events, it could easily have been assumed no tuna were landed during that particular survey.

### 2. Taxonomic accuracy

One of the most important facets of RFAMS was the ability to capture good biodiversity information from the catches. Such detailed information is currently not often captured in official catch statistics for tropical fisheries with the vast majority of minor catches lumped into “other fish” categories. However, to obtain this information, a sound knowledge of the landed biota is required. Obtaining accurate species level information is not always possible in such complicated multi-species and multi-gear fisheries without prior taxonomic effort. Nonetheless, family-level or group-level taxonomic data will still provide some useful biodiversity information. Basic taxonomic training is required for users of this method and identification tools such as line drawings and regional photographic guides are useful. In this study, material for a guide to Indonesian fishes was used [17]. Line drawings and keys for the family-level groupings which can be used in Indonesia and adjacent developing countries can be found in Appendix S1. Such guides should ideally be tailored to a specific region based on taxa likely to be encountered, but in their absence broader regional guides are still useful identification tools.

### 3. Acquisition of catch data

The use of the log-scale index of abundance for each taxonomic category was a rapid and simple method of recording abundance information from such large and complex catches. Capturing abundance indices is a minimum requirement when recording catch data in RFAMS. In the RFAMS conducted in Indonesia, it was also possible to record the bathome, fishing method and an estimated weight for each taxonomic category. This allows for a much more detailed assessment of catch data as it provides good information on the fisheries involved, as well as a volumetric estimate for each taxonomic category. However, it may not be possible to easily record all of these fields in much larger markets where volumes are much larger and/or harder to estimate. In these cases, an index of abundance may be the only field that can be recorded for each category.

In some instances, large quantities of fish were observed in the landing sites which also consisted of a diverse array of fish families and species. For example, the landings from trammel nets at Cilacap often contained several hundred kilos of small fishes (mostly <100 mm length). In such cases, sorting through the whole landing was not practical so the proportions of each family were obtained from a 10–20 kg subset of the catch. These proportions could then be used to estimate the total contribution of each of those families to the total landings present. Some rarer and less commercially important species may be missed in this exercise, but sub-sampling was needed to obtain useful information from these landings without sacrificing too much time examining the entire catch.

### 4. Important considerations of the RFAMS methodology

It is important to understand any limitations or other considerations of RFAMS so that the resulting analyses don't misinterpret the data collected or draw incorrect conclusions. The main considerations to be taken into account when using RFAMS are discussed below.

#### Number of data recorders required

While some landing sites are small and can easily be surveyed by one or two people, some landing sites are far larger and more complex and require a different approach. At the landing site of Muncar, the landings are dominated by very large quantities of sardines (*lemuru*) from the purse seine fishery and are spread out over a wide area. During peak fishing times, unloading occurs very quickly to enable the next boat access to the shore and trucks transporting these landings are constantly coming and going. To be able to adequately survey the daily landings at this site, at least four data recorders are required at the various landing areas.

#### Standing stock

Although some sites have a 100% turnover of landings from day to day, e.g. Tanjung Luar, Sadeng and Pacitan, other sites have a lower turnover of landings with a number of fish remaining in the market area for many days at a time, e.g. Kedonganan and Pelabuhanratu. This can present a problem if surveys are undertaken over consecutive days with some of the fish being recorded multiple times. In busy and complex fish markets, it can be difficult to discriminate freshly landed fish from ‘standing stock’ which may have been there for multiple days. It is not always possible to access this information from vendors.

#### Transhipment and translocation of catches

At some landing sites, produce is transported from other nearby locations either by sea or by land. For example, at Pelabuhanratu, large quantities of bigeyes (Priacanthidae) and moon fish (Menidae) are trucked to the market from East Java. Whenever possible, these components should be recorded separately. When dealing with multi-species and multi-gear fisheries, it is very difficult to obtain accurate capture locality information for all catch brought into the landing site or market. At some locations, catches from adjacent islands are landed separately from local catches, but once brought into the market area they are all combined. In order to accurately record capture fishing areas of all of the landings, a large number of data recorders positioned at all landing areas would be required which is not feasible in most cases.

#### Undertaking surveys for only short periods of time

Since surveys were conducted for short periods of time (usually 1–4 days) at each of the sites per survey trip, temporal variability in landings cannot be fully captured. Economic conditions at the time of a survey trip can influence landings. For example, at Cilacap, landings during some survey trips were much smaller than expected due to increased fuel prices which resulted in many fishers not going to sea but turning to earning income on land, e.g. agriculture. Periods of bad weather is another factor which can strongly influence the size of landings with rough sea conditions preventing smaller vessels from heading to sea to fish.

### 5. Multivariate analysis of the market survey data

**Figure 2:**
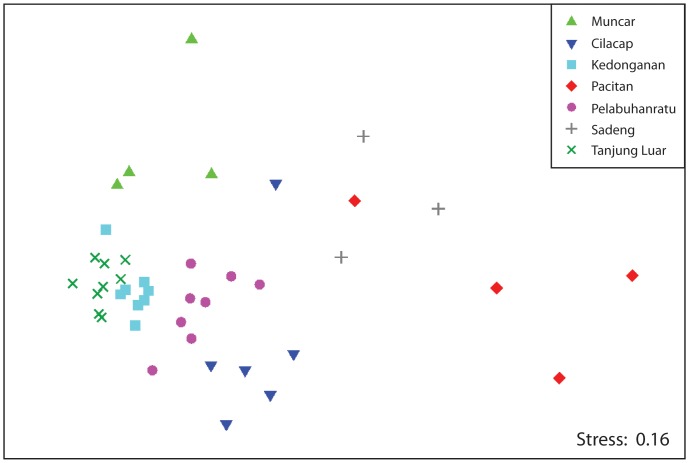
nMDS of family-level data at each site. Non-metric multidimensional scaling ordination of average daily estimated weights of each of the family-level taxonomic categories at each of the 7 sites on each of the survey trips.

When the average daily estimated weights of each family-level taxonomic category (e.g. Lutjanidae, Penaeidae) for each site on each trip were subjected to MDS ordination, the samples from each site formed relatively discrete groupings in most instances (Fig. 2). The samples from Kedonganan and Tanjung Luar formed the tightest groupings by far which is reflected by the low MVDISP values of 0.44 and 0.69, respectively. In comparison, the samples from Pacitan, Muncar and Sadeng were the most widely dispersed, reflected by high MVDISP values (1.68–1.75). A one-way ANOSIM showed that, overall, the samples for each site differed significantly from each other (*P*<0.01; *R*-statistic  = 0.823), and pairwise comparisons between sites showed that all sites differed significantly from each other (*P*<0.05; *R*-statistic>0.751), except Pacitan and Sadeng which did not differ significantly (*P*>0.05).

Since ANOSIM showed that the sites were significantly different from one another, SIMPER analyses on the same data set were also performed to determine the families which typified each site, and which families were most responsible for causing the difference between two sites (Table 1). It should be noted that while the most abundant families are often the most typifying and distinguishing groups in this type of analysis, they also need to be consistently typifying or distinguishing across all samples (e.g. one large catch of a group on only one occasion will rarely be an important family, even if its total biomass is very large). Table 1 summarises the families which typify each site, with Scombridae (tunas), Carangidae (trevallies) and Clupeidae (sardines) the most reoccurring families, and which distinguish each site.

**Table 1:** Results of the Similarity of Percentages (SIMPER) analyses of the daily estimated weight of family-level taxonomic categories at each site on each of the survey trips.

Survey site	Important families	Muncar	Cilacap	Kedonganan	Pacitan	Pelabuhanratu	Sadeng	Tanjung Luar
**Muncar**	1	*Clupeidae*						
	2	*Scombridae*						
	3	*Leiognathidae*						
**Cilacap**	1	Clupeidae	*Scombridae*					
	2	Scombridae	*squid*					
	3		*Penaeidae*					
**Kedonganan**	1	Clupeidae	Clupeidae	*Scombridae*				
	2	Lethrinidae	Lethrinidae	*Clupeidae*				
	3	Penaeidae	Lutjanidae	*Lutjanidae*				
**Pacitan**	1	Clupeidae	Scombridae	Clupeidae	*Scombridae*			
	2	Scombridae	squid	Lutjanidae	*Coryphaenidae*			
	3	Leiognathidae	Istiophoridae	Lethrinidae	*Carangidae*			
**Pelabuhanratu**	1	Clupeidae	Scombridae	Menidae	Clupeidae	*Scombridae*		
	2	Scombridae	Clupeidae	Bivalves	Trichiuridae	*Clupeidae*		
	3	Cnidaria	Menidae	Lethrinidae	Menidae	*Carangidae*		
**Sadeng**	1	Clupeidae	Scombridae	Clupeidae	-	Clupeidae	*Scombridae*	
	2	Leiognathidae	squid	Lethrinidae		Menidae	*Carangidae*	
	3	Cnidaria	Mobulidae	squid		Trichiuridae		
**Tanjung Luar**	1	Clupeidae	Scombridae	Mobulidae	Carcharhinidae	Trichiuridae	squid	*Scombridae*
	2	Mobulidae	Caesionidae	Carcharhinidae	Clupeidae	Menidae	Mobulidae	*Carcharhinidae*
	3	Cnidaria	Hemiramphidae	Clupeidae	Caesionidae	Carcharhinidae	Caesionidae	*Carangidae*

Italics indicate the families that typified a site; regular text are those families which distinguished two sites.

**Figure 3:**
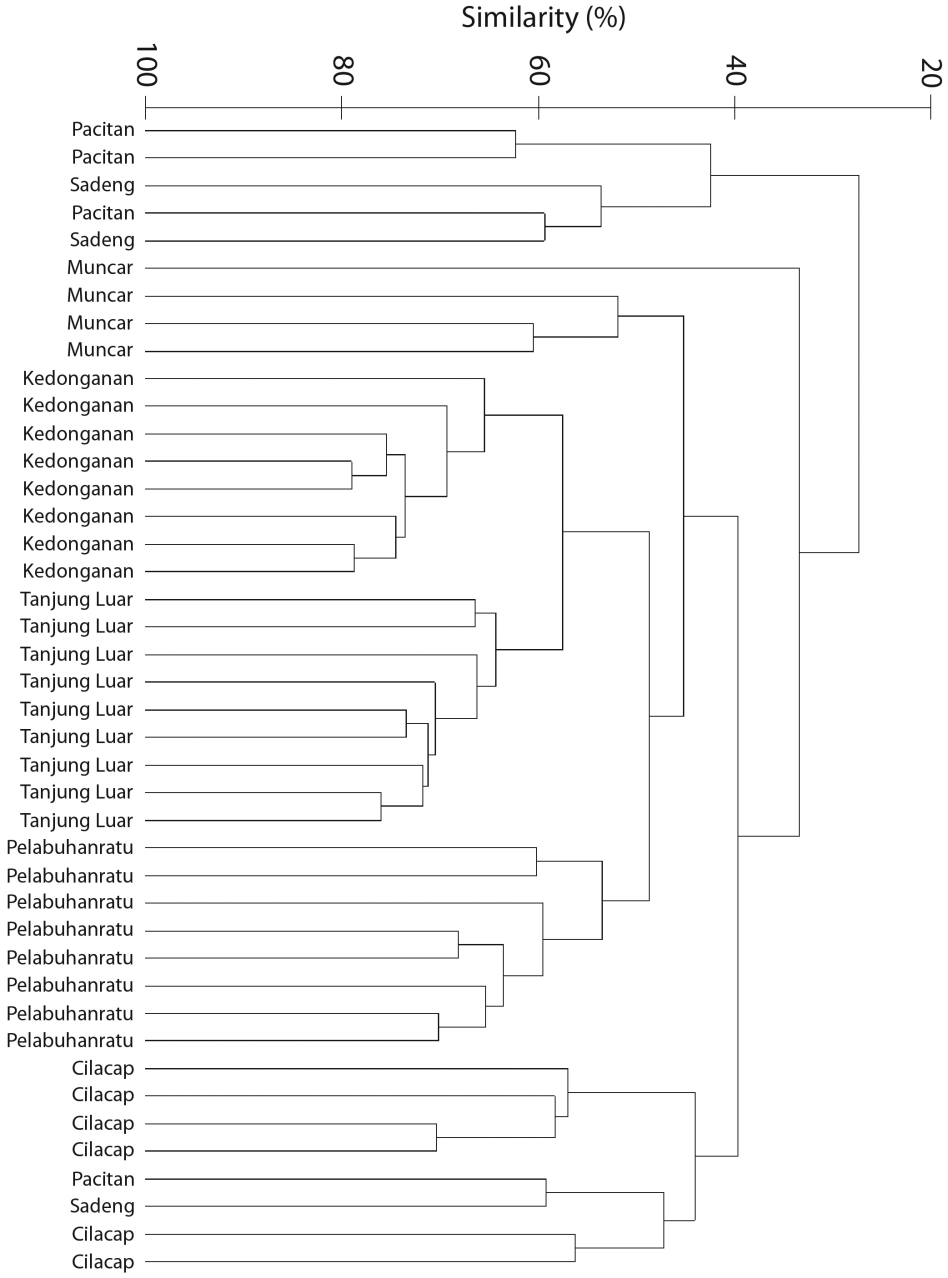
Cluster analysis of family-level data at each site. Average daily estimated weights of each of the family-level taxonomic categories at each of the 7 sites on each of the survey trips.

Cluster analysis of the same data also shows that each of the sites differ from one another in terms of catch composition (Fig. 3), but also allows a slightly better way of determining which sites are most different from others. For example, all but two of the Pacitan and Sadeng samples separate very quickly from the samples for the other sites at nearly 30% similarity, with the remaining two falling out with the Cilacap samples (reflecting the only two sampling occasions where large landings of scombrids were landed at these two sites). Pelabuhanratu and Muncar samples separate out at about 45–50% similarity from the Kedonganan and Tanjung Luar samples, which in turn separate out clearly from each other at close to 60% similarity (Fig. 3).

When the average daily estimated weights of each major taxonomic groups (e.g. Teleosts, Elasmobranchs) and also each functional group (e.g. reef fish – herbivore, inshore pelagics) for each site on each trip were subjected to MDS ordination, samples for each site formed relatively discrete groupings, particularly for functional groups. However, they were not as clearly delineated as in Fig. 2 when family-level categories were used. Although ANOSIM showed that in both cases, overall the samples for each site were significantly different, the *R*-statistic was much lower than when using family-level data (0.67–0.68 vs. 0.82). This suggests that obtaining more taxonomically accurate catch composition data allows for better characterisation of the markets.

**Figure 4:**
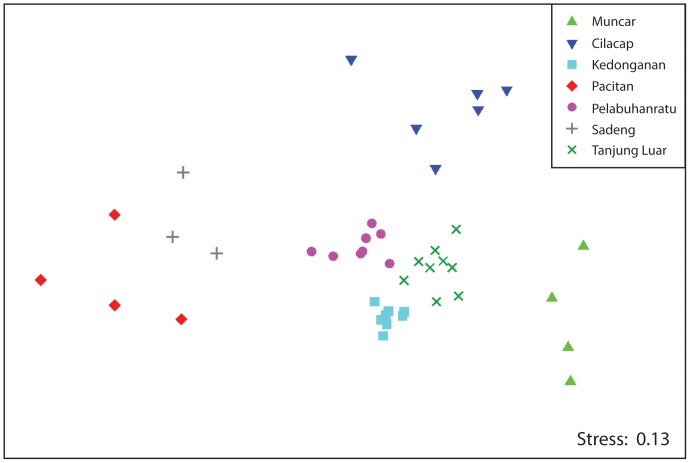
nMDS of bathome/gear type data at each site. Non-metric multidimensional scaling ordination of daily estimated weights of the landings of each of the bathome/gear types at each of the 7 sites on each survey trip.

When the average daily estimated weights of the landings from each of the major bathome/gear combinations (see Table S2) at each site on each survey trip were subjected to MDS ordination, the samples for each site formed very discrete groupings (Fig. 4). The Kedonganan samples were the most tightly formed groupings (MVDISP  = 0.48), indicating that across all of the survey trips, the landings within each of the bathome/gear combinations varied the least. Tanjung Luar and Pelabuhanratu samples also were relatively tight, with MVDISP values of 0.87 and 1.07, respectively. In comparison, the Pacitan and Muncar samples were the most dispersed (MVDISP  = 1.65 and 1.66), highlighting the wide variations in landings from various fisheries recorded at these sites. ANOSIM showed that sites were very significantly different from each other (*P*<0.001; *R*-statistic  = 0.87), which highlights that the gear used and bathomes fished are important determinants of the landings at each site. While this is to be expected, is does highlight that RFAMS is providing useful information on the multi-gear fisheries at these sites. SIMPER analysis of this data was also performed and those bathome/gear combinations that typified a site and those that distinguished between two sites are shown in Table 2.

**Table 2:** Results of the Similarity of Percentages (SIMPER) analyses of the average daily estimated weight of landings for each of the bathome/gear combinations (see Table S2) for each at each site on each of the survey trips.

Survey site	Important families	Muncar	Cilacap	Kedonganan	Pacitan	Pelabuhanratu	Sadeng	Tanjung Luar
**Muncar**	1	*InsPel:PurSei*						
**Cilacap**	1	InsPel:PurSei	*Oce:Lng*					
	2	Oce:Lng	*Oce:Gil*					
**Kedonganan**	1	InsPel:PurSei	Oce:Lng	*InsPel:Gil*				
	2	InsPel:Gil	CorRef:Hln	*CorRef:Hln*				
	3		InsPel:Gil	*Oce:Gil*				
**Pacitan**	1	InsPel:PurSei	Oce:Hln	InsPel:Gil	*Oce:Hln*			
	2	Oce:Hln	Oce:Lng	CorRef:Hln				
	3			Oce:Gil				
**Pelabuhanratu**	1	InsPel:PurSei	Oce:Hln	CorRef:Hln	InsPel:Gil	*Oce:Hln*		
	2	Oce:Hln	Oce:Lng	Oce:Hln	SloDem:Lng	*InsPel:Gil*		
**Sadeng**	1	InsPel:PurSei	Oce:Lng	InsPel:Gil	-	InsPel:Gil	*Oce:Hln*	
	2	Oce:Hln	Oce:Hln	CorRef:Hln		Oce:Gil		
	3			Oce:Gil		SloDem:Lng		
**Tanjung Luar**	1	InsPel:PurSei	Cor:Hln	Oce:Lng	InsPel:Gil	Oce:Hln	InsPel:Gil	*InsPel:Gil*
	2	InsPel:Gil	Oce:Lng	Est:Pot	Oce:Gil	Oce:Lng	Oce:Gil	*Oce:Gil*
	3		InsPel:Gil				Oce:Lng	*CorRef:Hln*

Italics indicate the bathome/gear combinations that typified a site; regular text are those that distinguished between two sites. Bathomes: CorRef  =  coral reef; Est  =  estuarine; InsPel  =  inshore pelagic; Oce  =  oceanic; SheDem  =  shelf demersal; SloDem  =  slope demersal. Gear: Gil  =  gillnet; Hln  =  handline; Lng  =  longline; Pot  =  trap or pot; PursSei  =  purse seine.

### 6. Conclusions

Despite the fact that a large proportion of the capture production of the world's fisheries comes from small-scale activities in developing countries, fisheries management in these regions are usually based on approaches and techniques from developed countries with little or no adaptation [18]. These approaches and the science supporting them have generally been applied to large stocks of few species, and are largely unsuitable for rich multispecies fisheries. A major impediment to fisheries science and management in developing countries is that fishery statistics are very difficult to collect due to a lack of well-trained staff and suitable data collection methods; this is further exacerbated as they are also typically diverse multi-gear and multi-species. In the case of Indonesia, a study commissioned by the Ministry of Fisheries [19] stated that the historical data collection methodology was obsolete and the information collected was of questionable quality. As a result, it was concluded that using this data to obtain Maximum Sustainable Yield (MSY) estimates is flawed.

The health of fisheries varies considerably throughout South-east Asia. Hence, there is an urgent need to improve catch statistics across the region. The region is too large and faunally diverse to adopt fishery independent assessment methods used elsewhere. Fish landings and markets offer a cost effective and viable data surrogate for wild catch information in developing countries, particularly those characterised by multi-gear and multi-species fisheries. RFAMS provides an important, standardised approach to characterising fish landing sites and markets in developing countries. This approach was proposed in the late 1990s during surveys of fish markets in the Philippines led by one of us (PL). The initial aim of this survey method was to determine whether it could be used to provide an indication of ecosystem and/or fishery health. For example, the species or taxonomic groups present or absent in a market are likely to partly reflect the level of fishing pressure in a particular area or fishery; for example, less commercially valuable species such as lizardfishes (Synodontidae) and ponyfishes (Leiognathidae) become increasingly more abundant in overfished demersal habitats at the expense of more valuable species such as snappers (Lutjanidae) and emperors (Lethrinidae) [20], [21]. Similarly, heavily fished areas/regions are likely to experience a trophic shift with decreasing proportions of top order predators and increasing proportions of lower trophic levels in the catch. This method alone will not provide the necessary information to determine ecosystem health, but will provide crucial baseline information required to support more focused investigations. This pilot study was subsequently adapted and implemented herein on a much larger scale in southern Indonesia.

The RFAMS approach is important for those contexts where there are no detailed and accurate data collection protocols. During this study, RFAMS was shown to effectively characterise the landings at each of the different landing sites and/or fish markets surveyed. This method allowed for data to be collected in a consistent, standardised manner across a wide range of sites, regardless of the size of the site and the scale of the landings. The multivariate analyses highlighted the way the resulting data can be used to characterise various sites based on catch data for each taxonomic category, fishing method and bathomic combination.

One of the main challenges of this methodology is the basic taxonomic knowledge required to allocate the landings to family-level (or proxy, e.g. squids) categories. However, multivariate analysis highlighted that the sites are better characterised from one another when using landings data for family-level categories, rather than functional groups (e.g. reef fish-herbivore) or major taxonomic groups (e.g. crustaceans). Species-level data should provide a better discrimination of sites, but such detailed information would be impossible to collect due to the high species richness at most landing sites. For example, at the Kedonganan fish market, an estimated average of 88 teleost species was observed each day. The speed of turnover at sites surveyed would also hinder species-level information to be collected. There is usually a constant movement of fish reaching and leaving these sites so data needs to be collected rapidly. Identification guides are needed for users of this method to be able to assign landings into the family-level categories. A line drawing guide, which includes basic identifying features, for Indonesian fish families is now available in [17]. This guide can be used effectively in adjacent regions in South-east Asia; although the species may differ, the families present will be very similar.

This novel approach for assessing landings at fish markets provides an important capacity building tool for developing countries. If used in the proposed standardised manner, the data from RFAMS can be used to ground-truth catch statistics from particular landing sites or fish markets. For example, at Tanjung Luar, the results from RFAMS for sharks were compared with local fisheries data and provincial (*Dinas Perikanan*) data for the times they overlapped. This comparison highlighted that the local fisheries data was similar to that collected in RFAMS, but the provincial data suggest far greater volumes and appears to be erroneous. Data from these surveys also provide important baseline information which can be used as a benchmark against which future changes in catch and market demographics can be assessed.

The data collected by RFAMS is also extremely useful for future studies in the regions surveyed. The data can be explored to show where particular groups are consistently landed and in what quantities, and the fishing methods dominating particular sites, etc. This can provide important information to direct future research and to allow comparisons to be made with other regions.

### 7. Recommendations and future efforts

The RFAMS method is an extremely useful tool for recording catches at fish landing sites in tropical developing countries where the catches are highly diverse with many fishing types involved. However, a potentially major issue is whether a large portion of the catches is being missed for any particular reason, e.g. catches by-passing the surveyed part of the landing site, sale direct from boats prior to catches entering landing site, etc. This needs to be determined on a case by case basis depending on the landing site being surveyed. Thus, one of the critical first steps of using this method is to develop a good understanding of the dynamics of the landing site or sites being surveyed. Using this method without understanding how a landing site operates can lead to significant underestimation of landings and potentially erroneous data being collected.

Landing sites can, and typically do, vary substantially in how they operate. Two landing sites which have similar catch composition and quantities may vary in other ways which can strongly influence the data being collected. A good example is timing of the landings. One landing site may have all catches landed in a two hour period while a second could have a similar period of landing followed by a second landing/unloading of catches much later in the day. This latter landing could simply be missed if it is assumed they operate at similar times. Similarly, one landing site may have certain components of the catch (e.g. high quality tuna of certain sizes) by-passing the typical landing site area. This process needs to be understood before data collection using the RFAMS method should be commenced.

The aim of this paper was to trial a rapid data collection method for complicated fisheries in data poor regions in a standardised manner. This study has demonstrated that RFAMS can be used to collect catch composition data in a standardised manner from different landing sites in Indonesia, but how this will be adopted by fisheries managers is the next major step. For the Indonesian context, RFAMS should ideally first be used at various fish landing sites and ports within each of the provinces for short period and compared with the fisheries statistics currently captured for the same period. This would highlight discrepancies in catch information and indicate where problems may be occurring. In this context, the RFAMS could be used as a quality control mechanism which could be extremely beneficial for local fisheries authorities. The adoption of RFAMS as a useful tool for helping to quality control and potentially standardising fisheries catch data collection would be highly beneficial to Indonesia and adjacent developing nations with large complex fisheries.

## Supporting Information

Table S1 **Functional groups.** List of the families (or species groups, e.g. squids) recorded during the RFAMS in Indonesia and the corresponding functional group each is allocated to.
(XLSX)

Table S2 **Major bathome and gear combinations recorded during this study.** Those combinations recorded less than 10 times during the study were allocated to 'Rare'. Abbreviations used in MDS analyses are also included.
(XLSX)

Datasheet S1 **The Rapid Fisheries Assessment by Market Survey (RFAMS) data sheets.** Used to capture the daily landings data and landing site characteristics.
(XLS)

Appendix S1 **Line drawing guide.** Guide and keys to the marine, family-level categories used in RFAMS. Suitable for Indonesia and adjacent developing countries.
(PDF)
