# Supplementary material for: Rapid Fishery Assessment by Market Survey (RFAMS) – An Improved Rapid-Assessment Approach to Characterising Fish Landings in Developing Countries
Source: PLoS One. 2014 Oct 2;9(10):e109182. doi: 10.1371/journal.pone.0109182 (PMC4183571; doi:10.1371/journal.pone.0109182)
Supplement: Appendix S1 — Line drawing guide. Guide and keys to the marine, family-level categories used in RFAMS. Suitable for Indonesia and adjacent developing countries. (PDF) [file pone.0109182.s004.pdf]

Appendix S1

Line drawing guide.

Guide and keys to the marine, family-level categories used in RFAMS. Suitable for Indonesia and adjacent developing countries.

Crustaceans
(crays, crabs, prawns, shrimps)

Main groups recorded in RFAMS

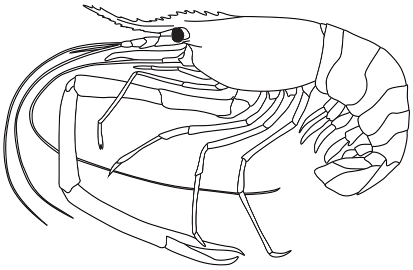

Palaemonidae (palaemonid shrimps)

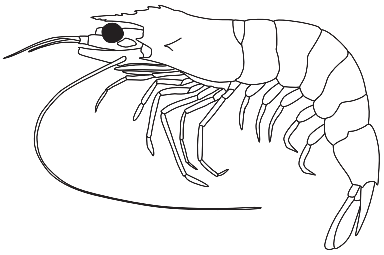

Penaeidae (penaeid shrimps, prawns)

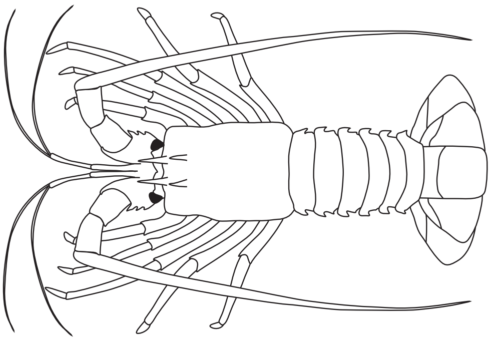

Palinuridae (spiny lobsters)

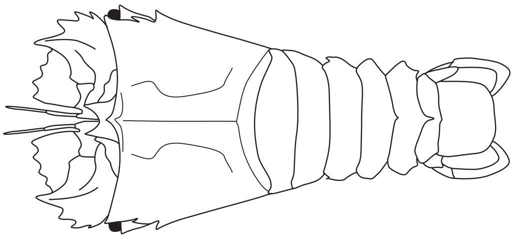

Scyllaridae (slipper lobsters)

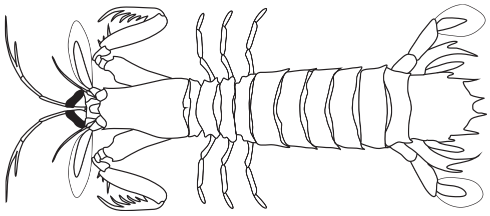

Stomatopods (mantis shrimp, killer prawns)

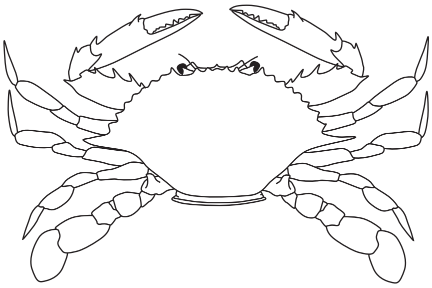

Portunidae (swimming crabs, mud crabs)

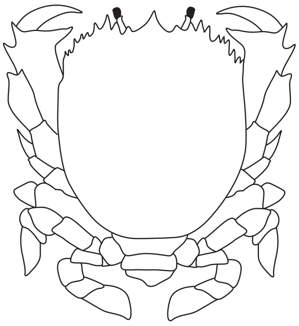

Raninidae (spanner crabs)

Molluscs

Main groups recorded in RFAMS

Cephalopods (squid, octopus, cuttlefish, nautilus)

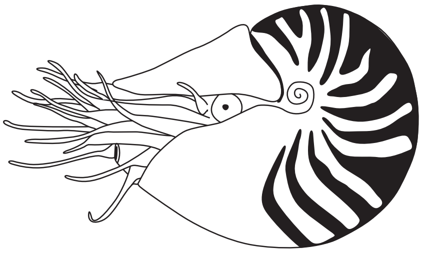

Nautilidae (nautilus)

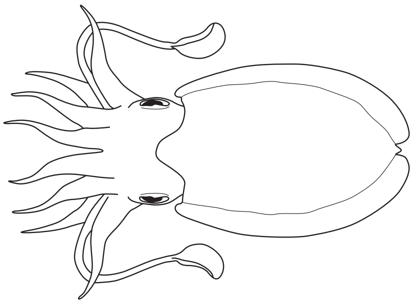

Sepiidae (cuttlefishes)

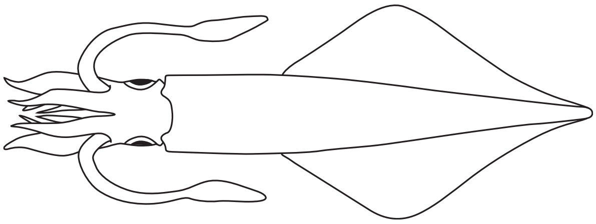

Squids (all squids combined)

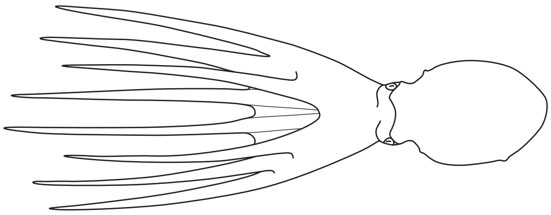

Octopodidae (benthic octopuses)

Other molluscs

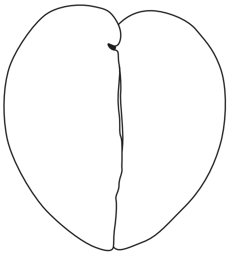

Typical bivalves (e.g. cockles, clams, mussels, oysters)

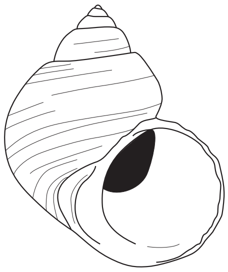

Typical gastropods (e.g. limpets, abalone, turban shells, periwinkles, cowries)

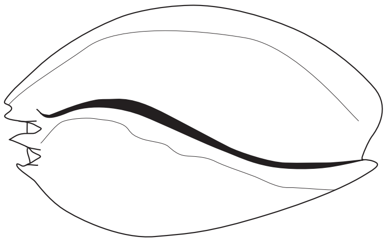

Volutidae (volutes, baler shell)

Other organisms

Main groups recorded in RFAMS

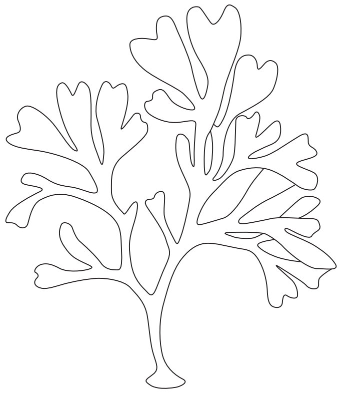

Algae (all types of seaweeds)

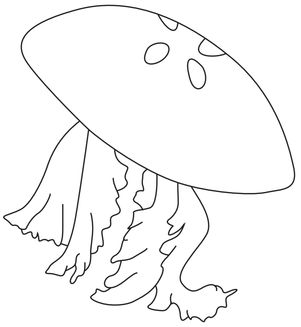

Cnidaria (jellyfishes)

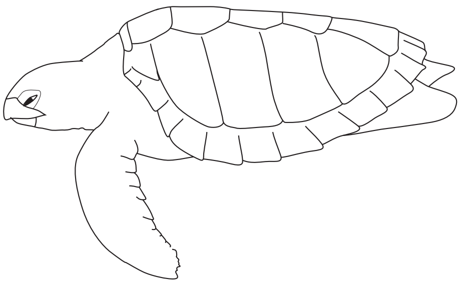

Cheloniidae (sea turtles)

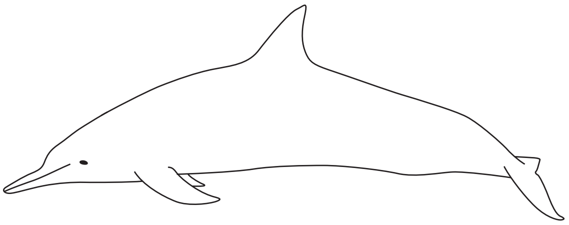

Delphinidae (true dolphins)

Chondrichthyans
(sharks, rays, chimaeras)

Key to the main families in the area

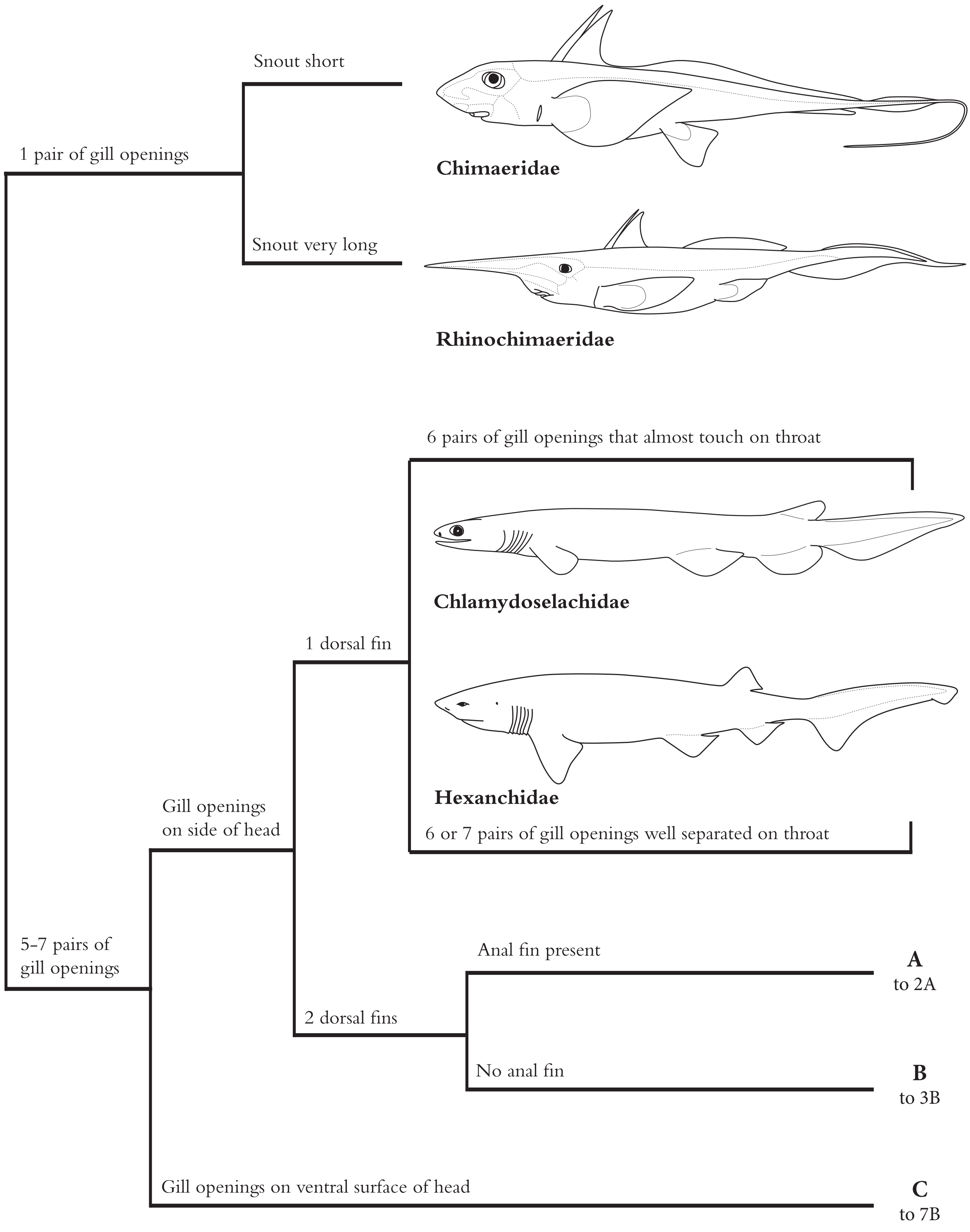

A
from 1A

Snout not blade-like with lateral teeth

Snout greatly elongate, blade-like, with numerous, sharp, lateral teeth and a pair of long barbels

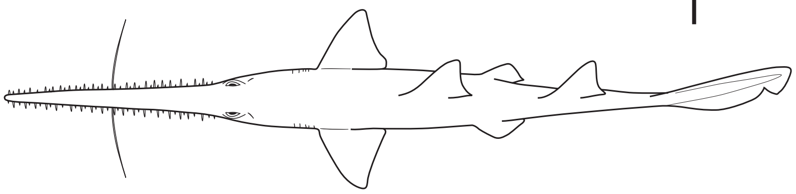

Pristiophoridae

Body not strongly depressed; pectoral fins small to moderate in size

Body strongly depressed; pectoral fins very large and broad

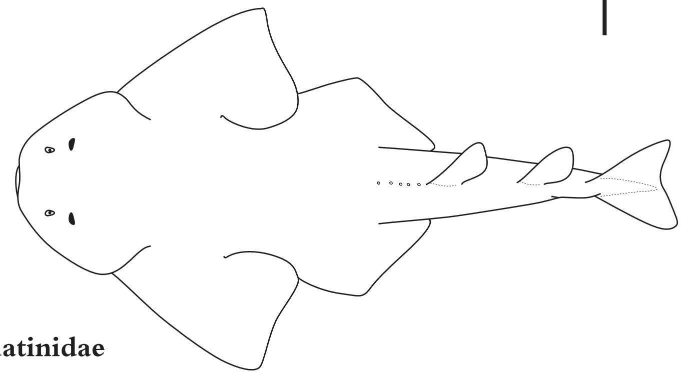

Squatinidae

Denticles not extremely large; 1st dorsal fin closer to pectoral fins

Denticles extremely large; 1st dorsal fin origin over pelvic-fin bases

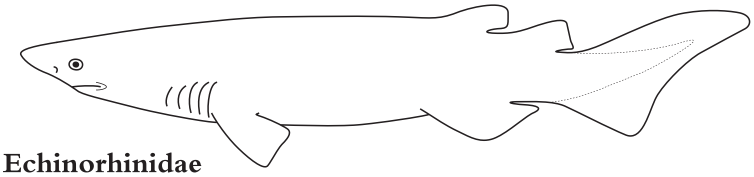

Echinorhinidae

No strong keel on caudal peduncle; a terminal lobe on caudal fin; spines present or absent

Strong keel on caudal peduncle; no terminal lobe on caudal fin; strong dorsal spines present

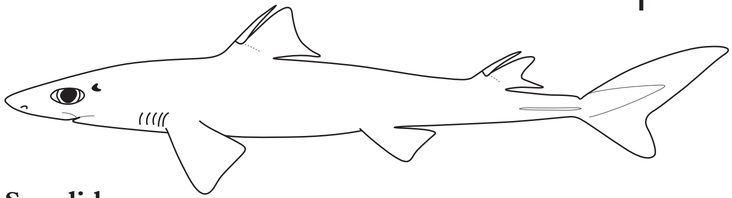

Squalidae

A
to 3A

A
from 2A

Upper teeth without lateral cusplets; if present, dorsal-fin spines often more even in height

Upper teeth with strong lateral cusplets; 2nd dorsal-fin spine much longer than 1st

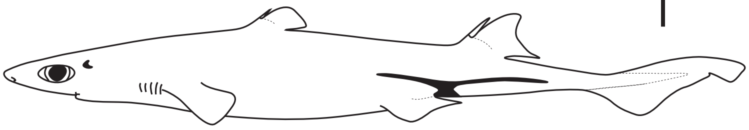

Etmopteridae

Dorsal-fin spines absent or very short

Dorsal-fin spines prominent

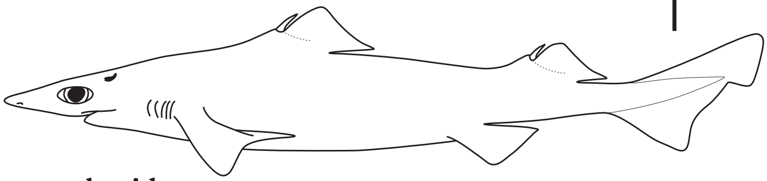

Centrophoridae

Head quite broad and somewhat flattened

Head narrow, rounded to conical

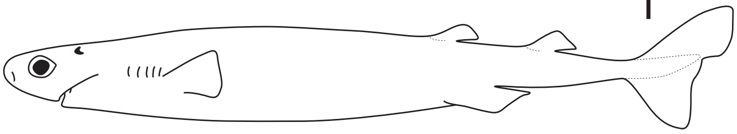

Somniosidae

Dalatiidae

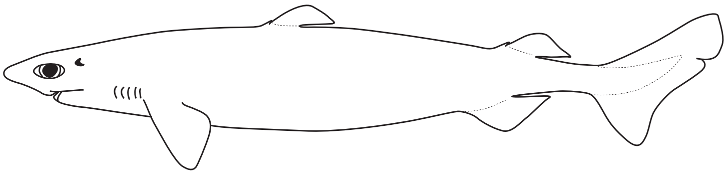

B
from 1B

Caudal fin nearly as long or longer than body

Body yellow with numerous spots; strong ridges on sides

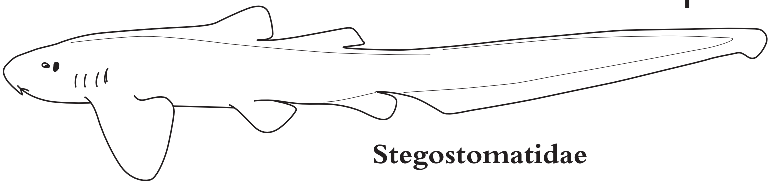

Stegostomatidae

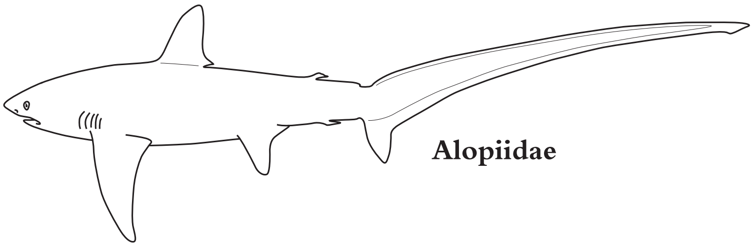

Alopiidae

Body bluish grey without spots; no ridges on sides

Caudal fin much less than half body length

A
to 4A

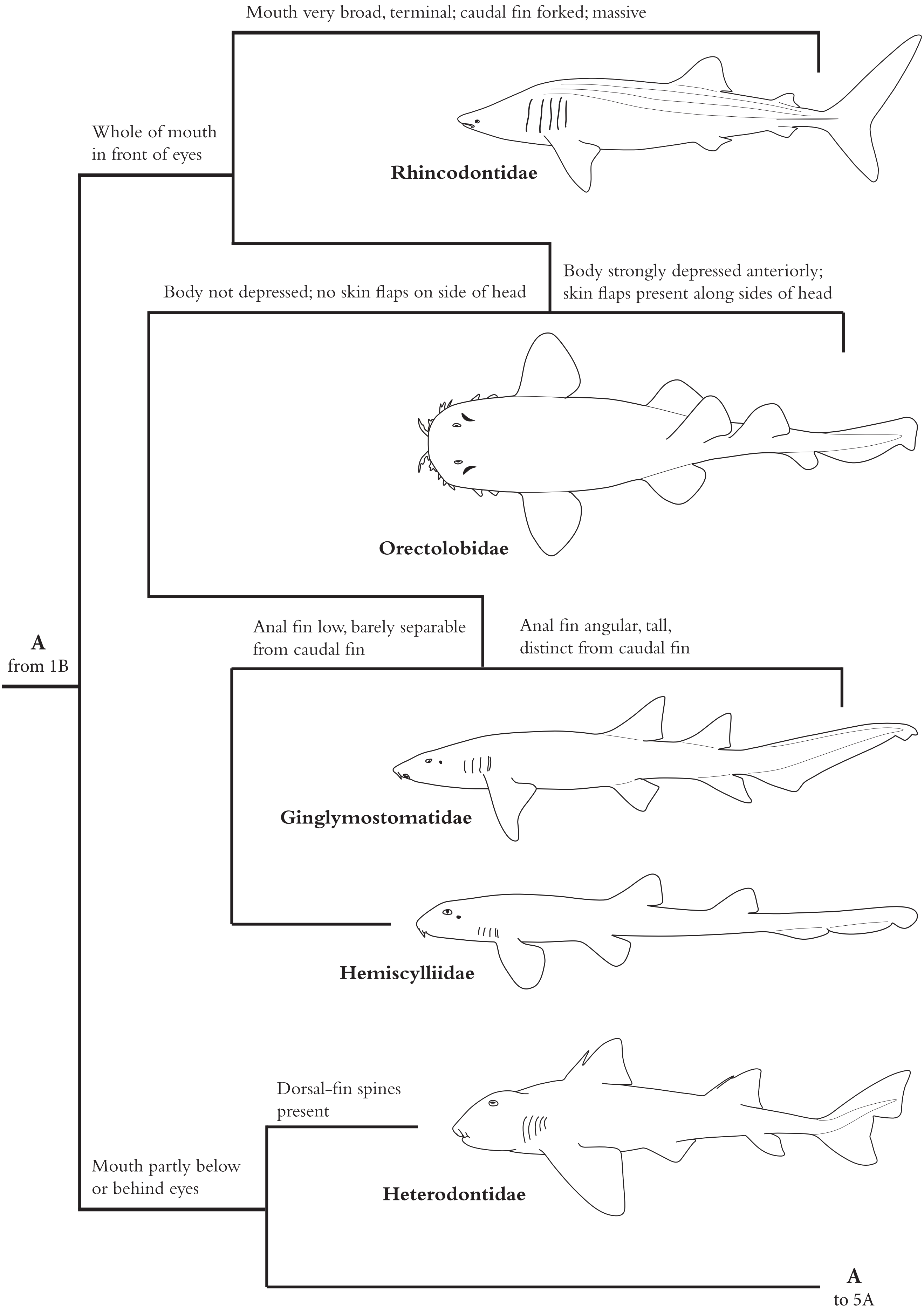
Mouth very broad, terminal; caudal fin forked; massive
Whole of mouth
in front of eyes
Rhincodontidae
Body strongly depressed anteriorly;
skin flaps present along sides of head
Body not depressed; no skin flaps on side of head
Orectolobidae
Anal fin low, barely separable
from caudal fin
Anal fin angular, tall,
distinct from caudal fin
A
from 1B
Ginglymostomatidae
Hemiscylliidae
Dorsal-fin spines
present
Mouth partly below
or behind eyes
Heterodontidae
A
to 5A

A
from 5A

Head without lateral expansions

Head with broad, lateral expansions, hammer-shaped

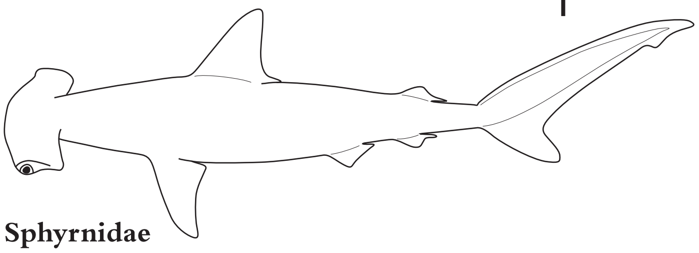

Sphyrnidae

Upper part of snout not greatly elongate; jaws not protrusible

Upper part of snout greatly elongate; jaws highly protrusible

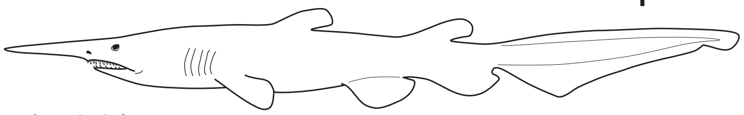

Mitsukurinidae

Mouth not huge and subterminal

Mouth huge and at front of head

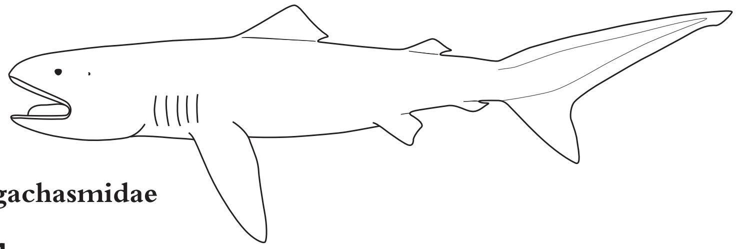

Megachasmidae

1st dorsal-fin origin in well in front of pelvic-fin origin

1st dorsal-fin origin well behind pelvic-fin origin

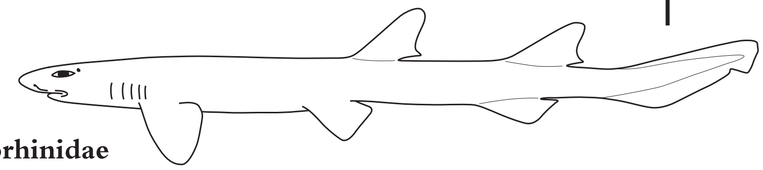

Scyliorhinidae

Caudal fin lunate, upper and lower lobes of similar length

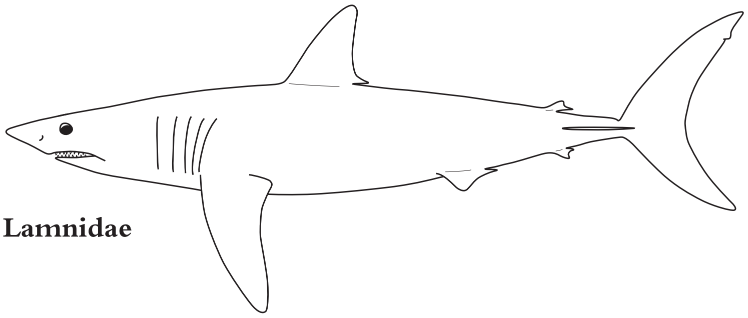

Lamnidae

A
to 6A

A
from 5A

Eyes smaller; gill slits not extending onto dorsal surface

Eyes very large; gill slits very large, extending onto dorsal surface of head

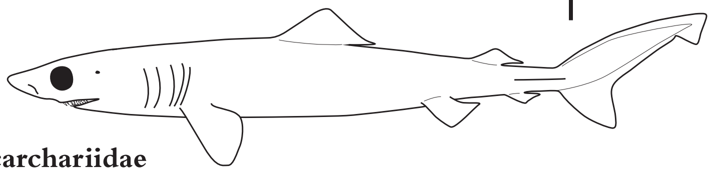

Pseudocarchariidae

Eyelid capable of closing over eye (nictitating)

Eyelid fixed, not capable of closing over eye

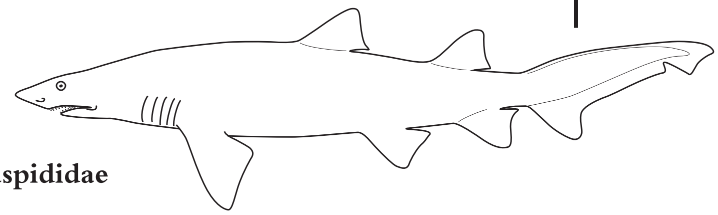

Odontaspididae

Precaudal pits present at origin of caudal fin

Spiracles absent (except in tiger shark *Galeocerdo cuvier*)

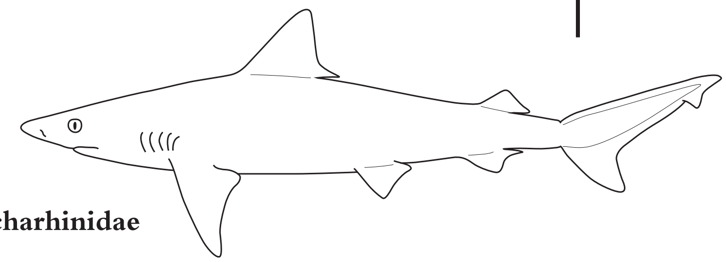

Carcharhinidae

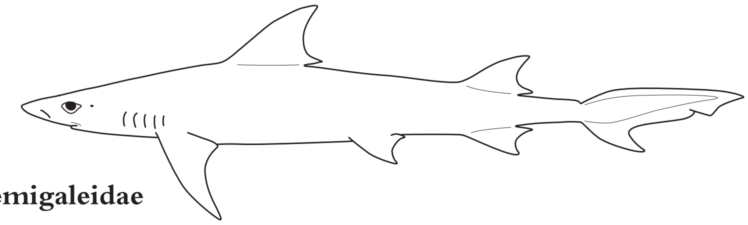

Hemigaleidae

Small, but distinct spiracles present

Precaudal pits absent

Teeth small, numerous (>100 rows)

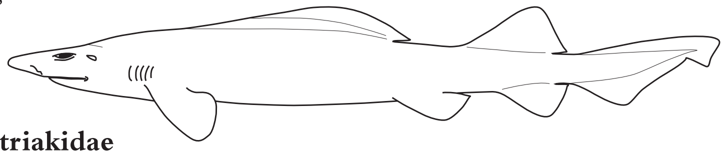

Pseudotriakidae

Teeth larger, much less than 100 rows

A
to 7A

A
from 6B

Labial furrows at corners of mouth relatively long; ventral caudal lobe short but distinct

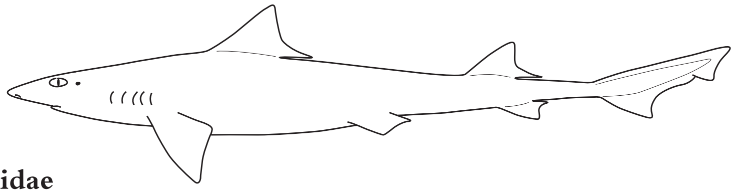

Triakidae

Labial furrows at corners of mouth short or absent; ventral caudal lobe barely evident

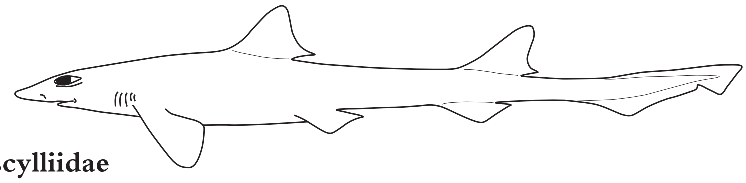

Proscylliidae

B
from 1C

Snout not saw-like

Snout greatly elongated, blade-like with numerous sharp lateral teeth

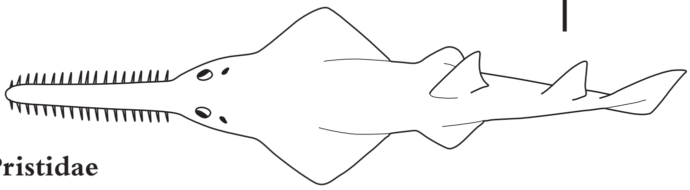

Pristidae

2 prominent dorsal fins present; origin of 1st dorsal fin closer to pelvic fins than tip of tail

Body hard to touch, rough

A
to 8A

Body soft to the touch, slimy

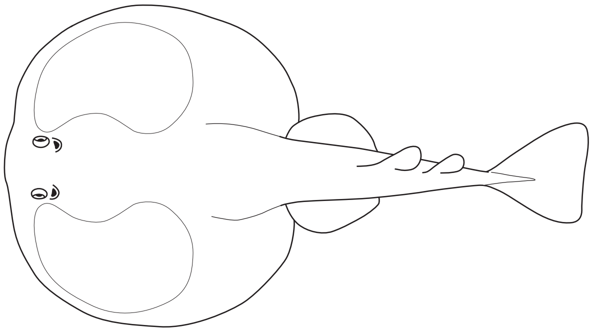

Mouth broadly arched; 1st dorsal fin larger than 2nd

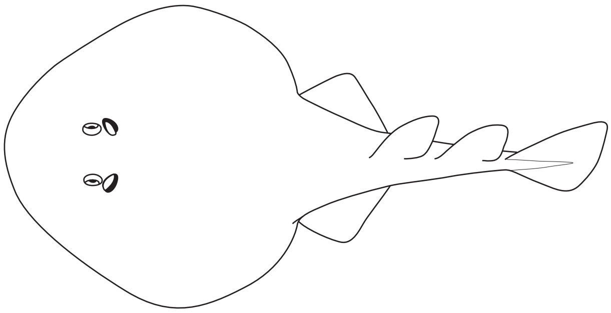

Mouth almost straight; dorsal fins similar in size larger than 2nd

0-2 dorsal fins; when 2 dorsal fins present, origin of 1st closer to tip of tail than pelvic fins

B
to 8B

A
from 7A

Snout triangular, pointed

Snout broadly rounded, not pointed

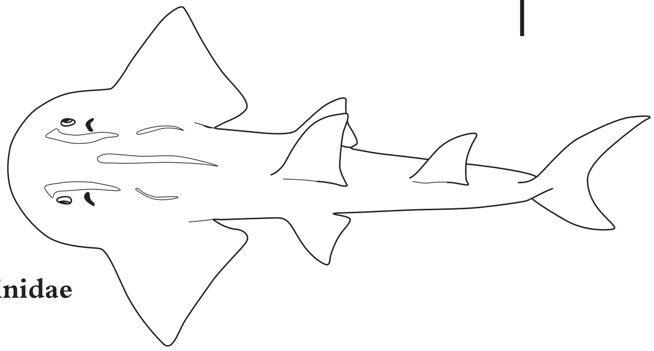

Rhinidae

Caudal fin without a distinct lower lobe

Caudal fin with a distinct lower lobe

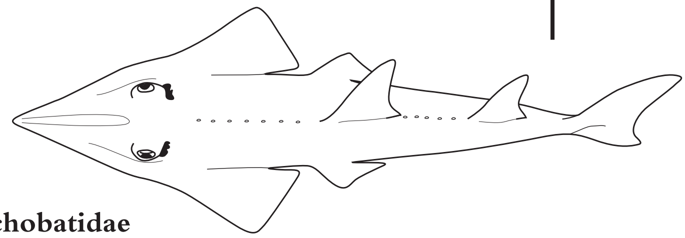

Rhynchobatidae

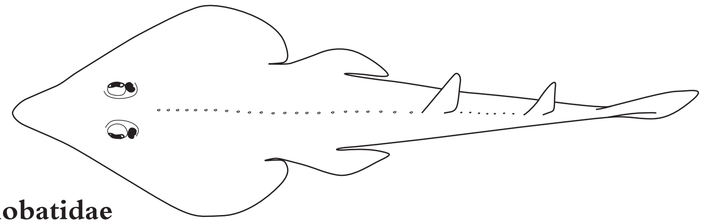

Rhinobatidae

B
from 7B

6 pairs of gill openings

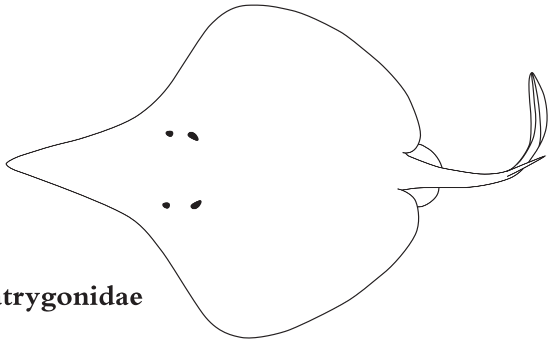

Hexatrygonidae

5 pairs of gill openings

Head elevated and laterally demarked from disc; eyes lateral on head — **A** to 9A

Head not elevated, not clearly separable from disc; eyes dorsolateral on head — **B** to 9B

A
from 8A

- A pair of paddle-like flaps at front of head

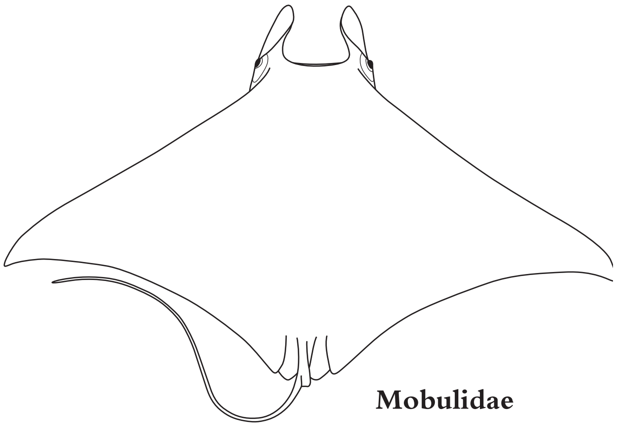

Mobulidae

- No flaps at front of head
  - Front margin of snout with a deep central notch

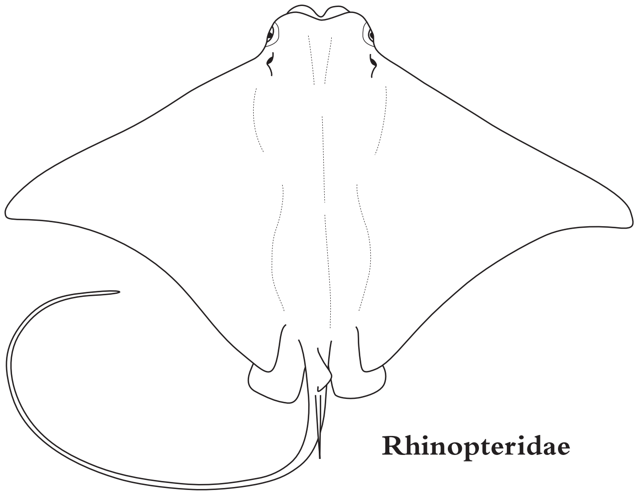

Rhinopteridae

  - Front margin of snout without a central notch

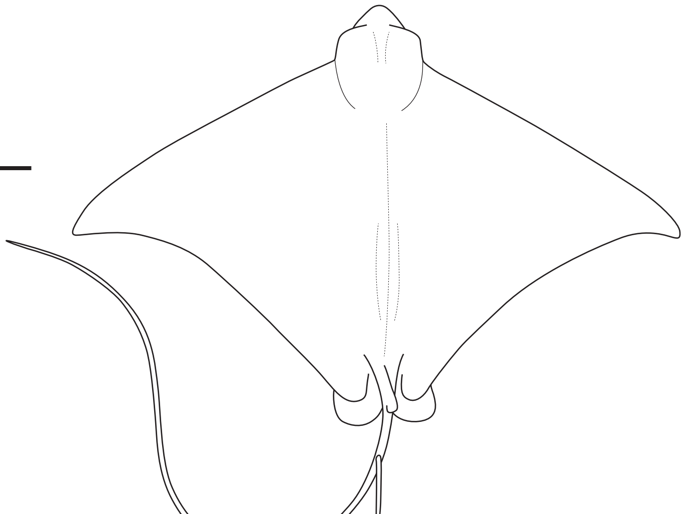

Myliobatidae

B
from 8B

- Pelvic fin divided into 2 distinct lobes — **A** to 10A
- Pelvic fin not divided into 2 lobes
  - Caudal fin present — **B** to 10B
  - No caudal fin — **C** to 11A

A
from 8A

Tail very thin and filamentous

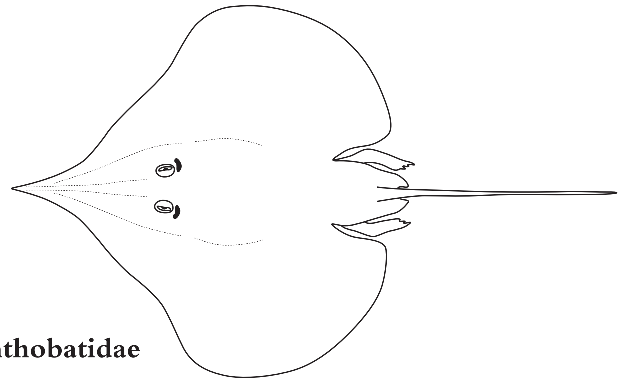

Anacanthobatidae

Tail thicker, not filamentous

Snout soft with thin and flexible rostral cartilage

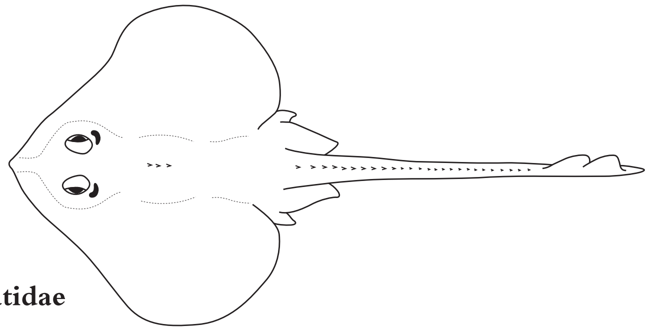

Arhynchobatidae

Snout firm with stiff rostral cartilage

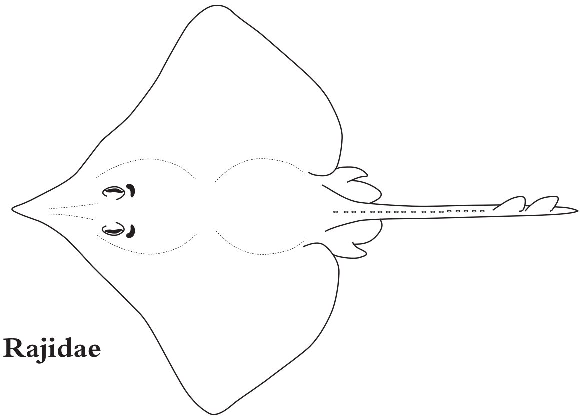

Rajidae

B
from 9B

No dorsal fins; strong stinging spine present

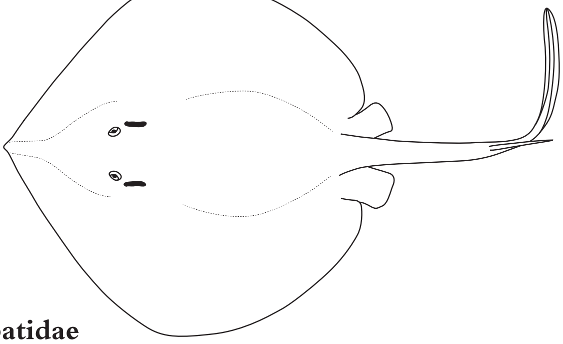

Plesiobatidae

0 or 1 dorsal fins; no stinging spines

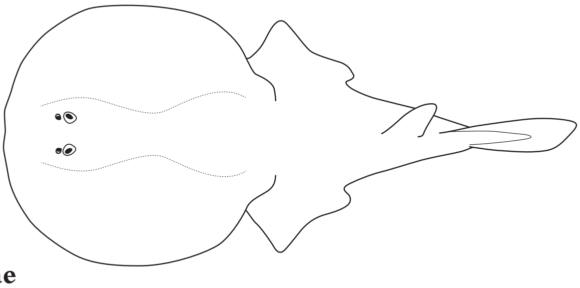

Narkidae

A
from 9C

Disc very broad; tail short and thin

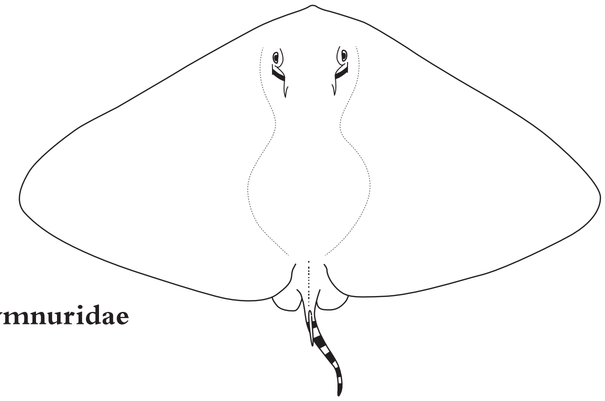

Gymnuridae

Disc not laterally expanded; tail longer

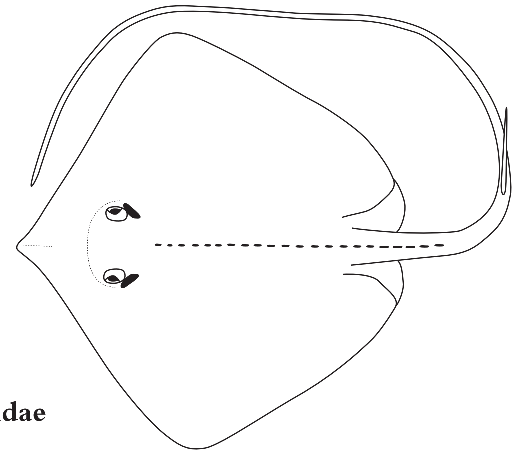

Dasyatidae

Teleosts (bony fishes)

Key to the main families in the area

- Body encased in bony plates
  - Snout tubular
    - Body extremely compressed, blade-like

      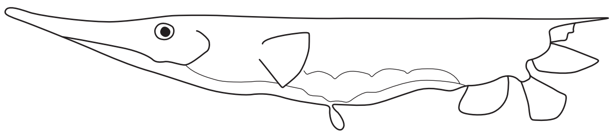

      Centriscidae
    - Body not extremely compressed

      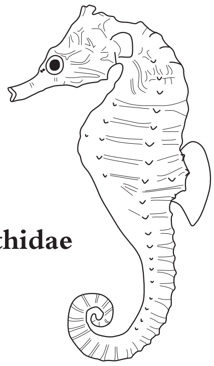

      Syngnathidae
  - Snout not tubular
    - 2 separate dorsal fins

      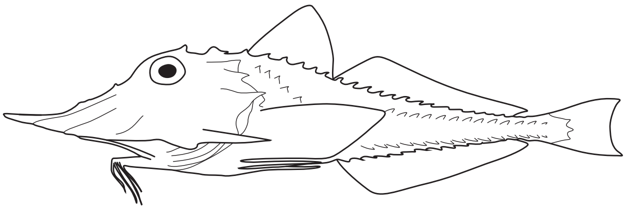

      Peristediidae
    - A single dorsal fin

      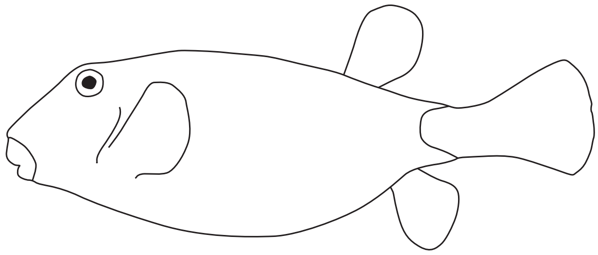

      Ostraciidae
- Body not encased in bony plates
  - Elongate bill-like or beak-like jaws — **A** to 2A
  - Jaws not bill-like or beak-like
    - Body strongly flattened; eyes on one side of head — **B** to 2B
    - Body not strongly flattened; an eye on each side of head — **C** to 3B

A
from 1A

Only lower jaw greatly elongate

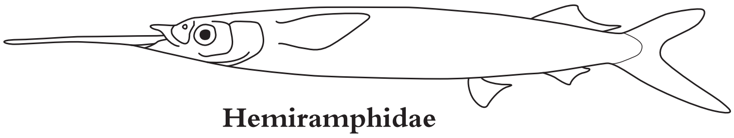

Hemiramphidae

Both jaws greatly elongate

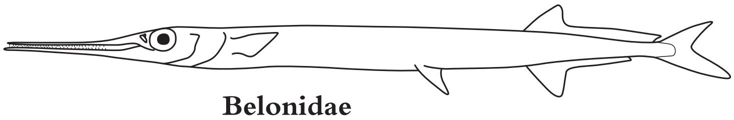

Belonidae

Only upper jaw greatly elongate

1 keel on each side of caudal peduncle; bill flat in cross-section

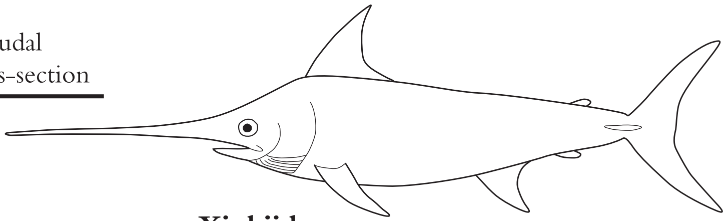

Xiphiidae

2 keels on each side of caudal peduncle; bill round in cross-section

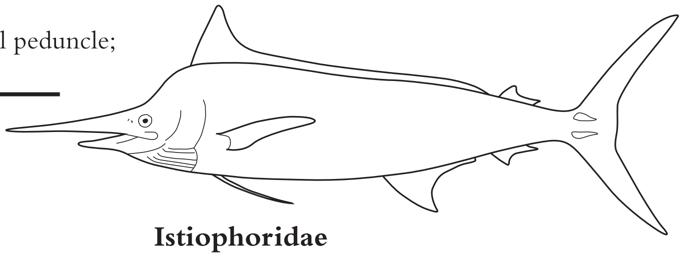

Istiophoridae

B
from 1B

Teeth very large, very distinct

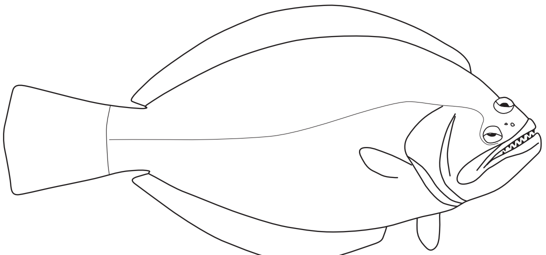

Psettodidae

Teeth small, usually not

Eyes on right side of body

Margin of preopercle not free, embedded in skin

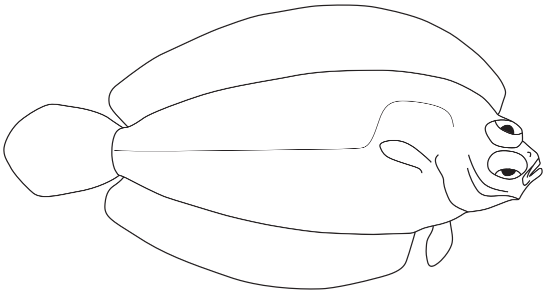

Pleuronectidae

Margin of preopercle free and distinct

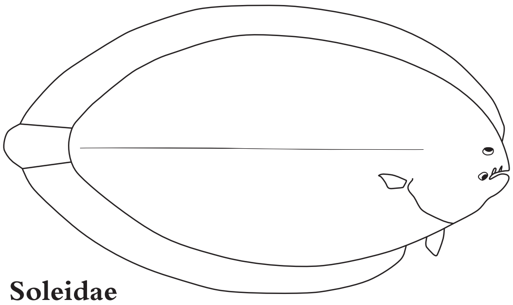

Soleidae

Eyes on left side of body

A
to 3A

A
from 2A

Pectoral fins absent

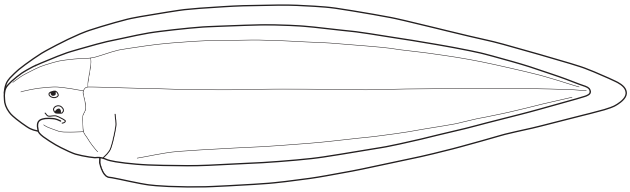

Cynoglossidae

Pectoral fins present

Lateral line present below lower eye

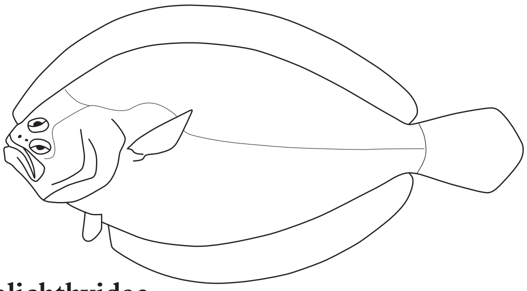

Paralichthyidae

No lateral line below lower eye

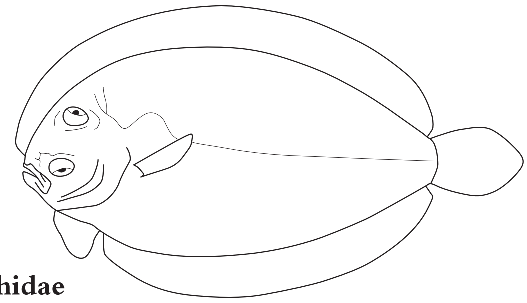

Bothidae

B
from 1C

Tail very elongate, ribbon-like or eel-like with no distinct (except in some Trachipteridae) caudal fin

1 short dorsal fin; snout gelatinous

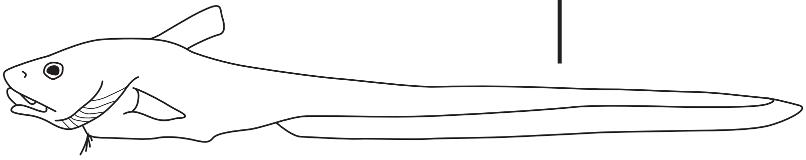

Ateleopodidae

1 long dorsal fin or 2 dorsal fins

2 dorsal fins: 1st short, tall; 2nd long, low

4 pairs of barbels on head; no scales

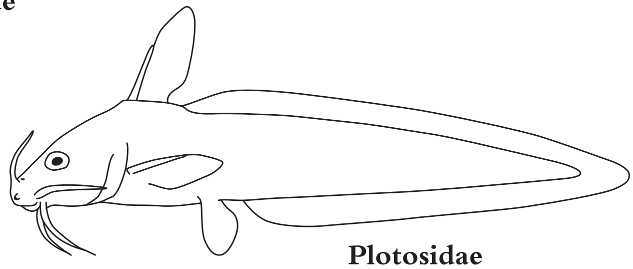

Plotosidae

often 1 barbel on chin; large scales present

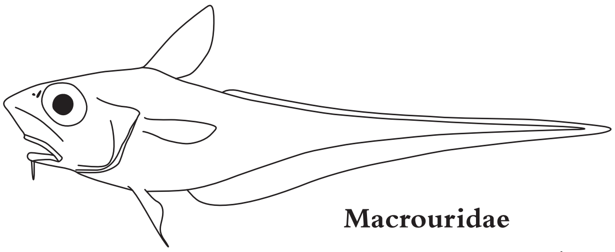

Macrouridae

Dorsal fin not as above — **A** to 4A

Tail not ribbon-like or eel-like; caudal fin distinct — **B** to 6A

External gill opening a large bony operculum

Anal fin distinct

Anal fin absent or reduced to short spinules

Skin covered with bony, raised tubercles

Skin smooth, without scales

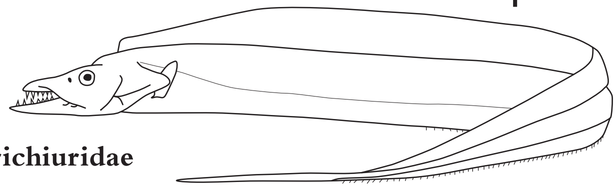

Trichiuridae

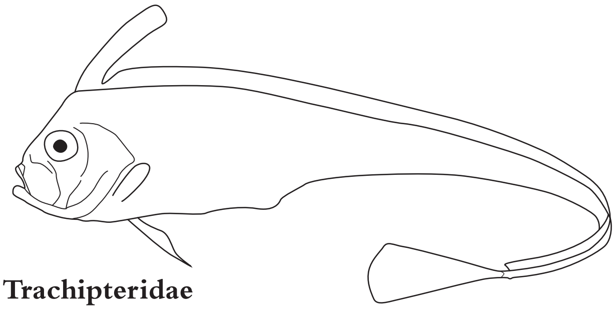

Trachipteridae

A
from 3A

Pelvic fins absent or consisting of 1 or 2 separate rays

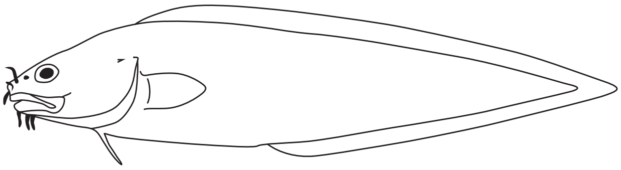

Ophidiidae

Body very elongate, with dark bands

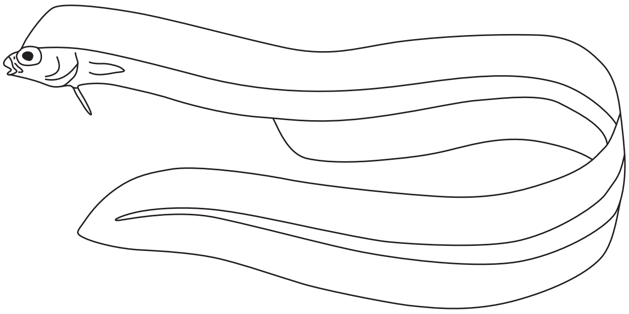

Pelvic fins present

Blenniidae genus *Xiphasia*

Body elongate, orange to reddish

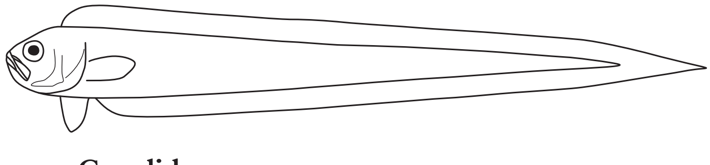

Cepolidae

External gill opening a small, fleshy opening

A
to 5A

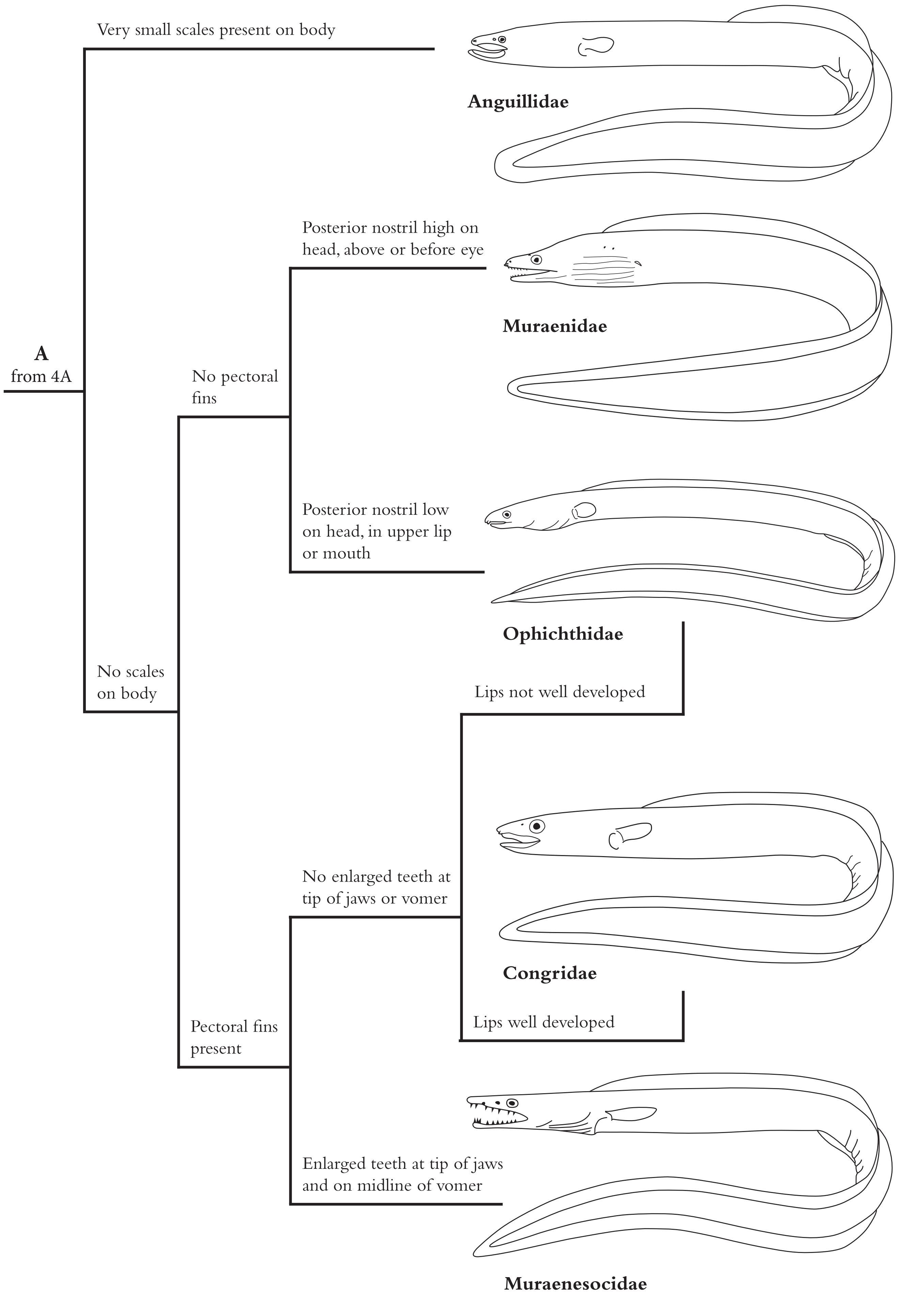
Very small scales present on body
Anguillidae
Posterior nostril high on head, above or before eye
Muraenidae
A
from 4A
No pectoral fins
Posterior nostril low on head, in upper lip or mouth
Ophichthidae
No scales on body
Lips not well developed
No enlarged teeth at tip of jaws or vomer
Congridae
Lips well developed
Pectoral fins present
Enlarged teeth at tip of jaws and on midline of vomer
Muraenesocidae

A
from 3B

- Pectoral fins greatly enlarged, wing-like
  - 1 dorsal fin

    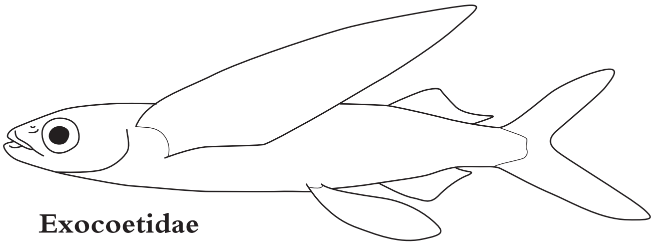

    Exocoetidae
  - 2 dorsal fins and 2 free dorsal-spines anteriorly

    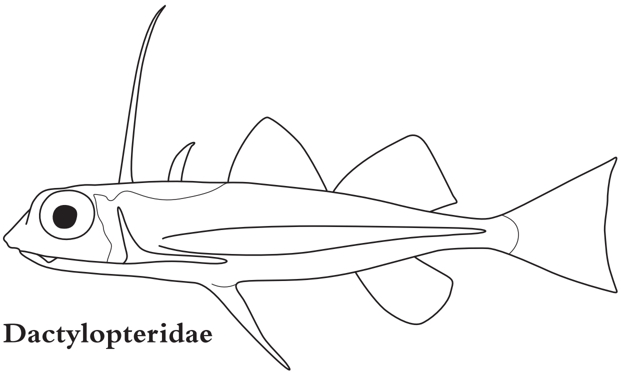

    Dactylopteridae
- Pectoral fins not wing-like
  - 1st dorsal fin modified into a sucking disc on top of head

    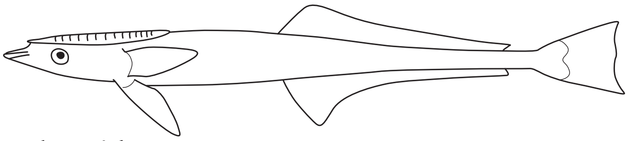

    Echeneidae
  - 1 short dorsal fin
    - Snout tubular, flute-like

      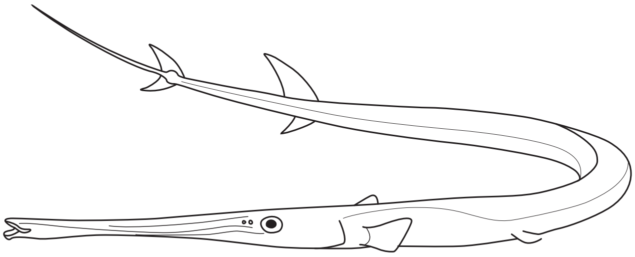

      Fistulariidae
    - Snout not flute-like
      - Caudal fin truncate and rudder-like; body very deep

        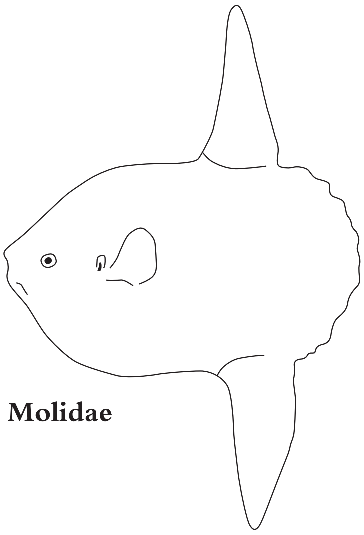

        Molidae
      - Caudal fin not rudder like; body not very deep — **A** to 7A
  - 1 short dorsal fin with a small ray-less adipose fin posteriorly — **B** to 9A
  - 1 long dorsal fin or 2 separate dorsal fins — **C** to 9B

A
from 6A

Caudal fin truncate or rounded; belly inflatable

Body covered in many sharp spines

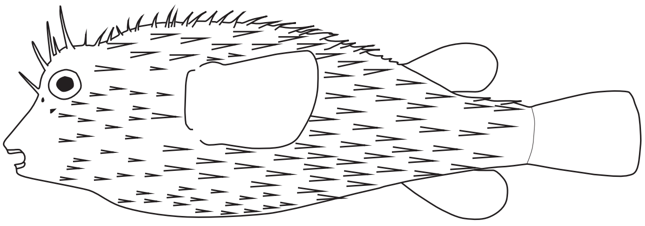

Diodontidae

Body without sharp spines

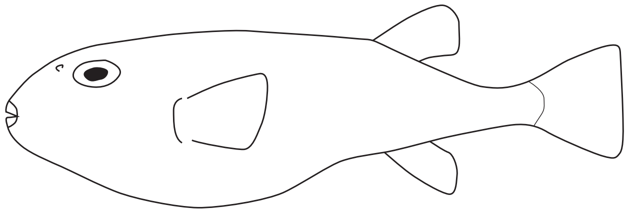

Tetraodontidae

Caudal fin forked; belly not inflatable

Caudal fin shallowly forked; lateral line extends onto caudal fin

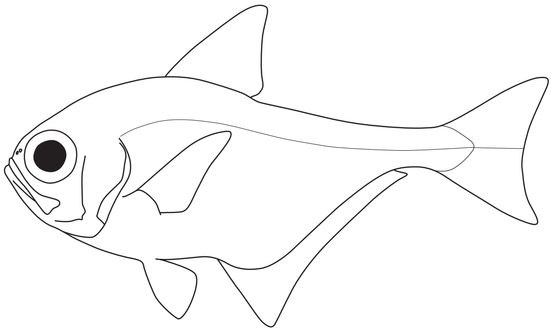

Pempheridae

Caudal fin deeply forked; lateral line not on caudal fin

Body bright red

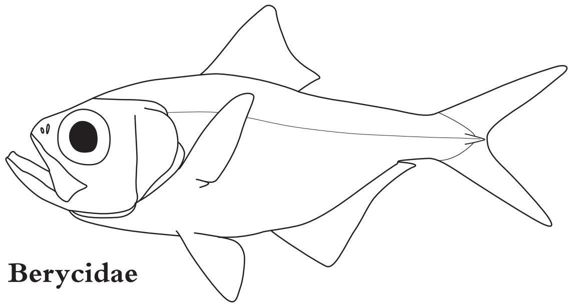

Berycidae

Body mostly silvery

Dorsal fin located set far back on body; teeth fang-like

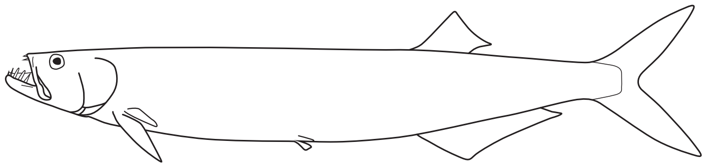

Chirocentridae

Dorsal fin located above middle of body; teeth not fang-like

A
to 8A

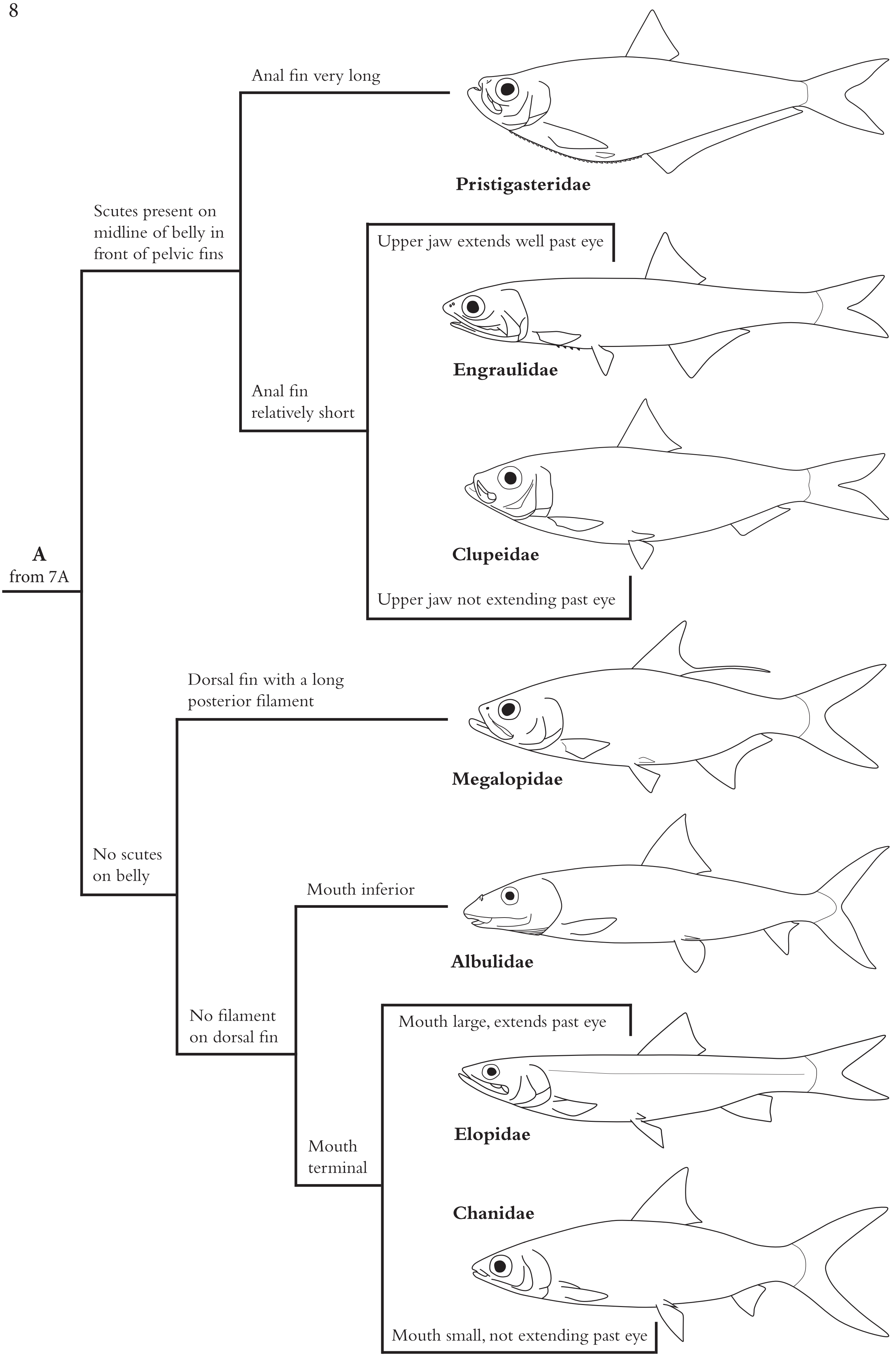
A
from 7A
Scutes present on midline of belly in front of pelvic fins
Anal fin very long
Pristigasteridae
Anal fin relatively short
Upper jaw extends well past eye
Engraulidae
Clupeidae
Upper jaw not extending past eye
No scutes on belly
Dorsal fin with a long posterior filament
Megalopidae
No filament on dorsal fin
Mouth inferior
Albulidae
Mouth terminal
Mouth large, extends past eye
Elopidae
Chanidae
Mouth small, not extending past eye

A
from 6B

Barbels present around mouth

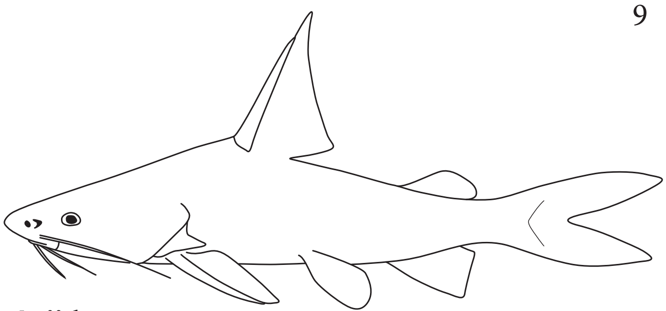

Ariidae

No barbels on head

Snout elongate

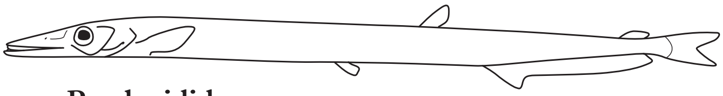

Paralepididae

Snout not elongate

Light organs present on body

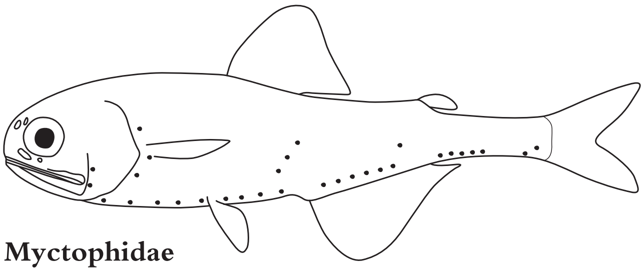

Myctophidae

No light organs on body

Upper jaw not extending to end of eye

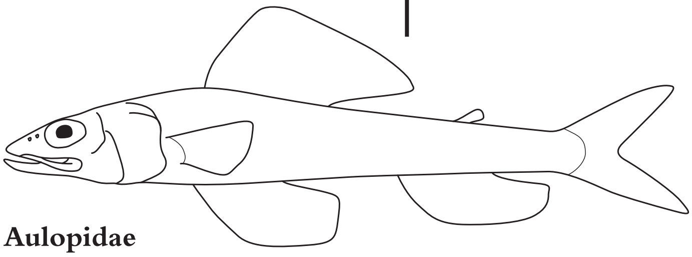

Aulopidae

Upper jaw extending well past eye

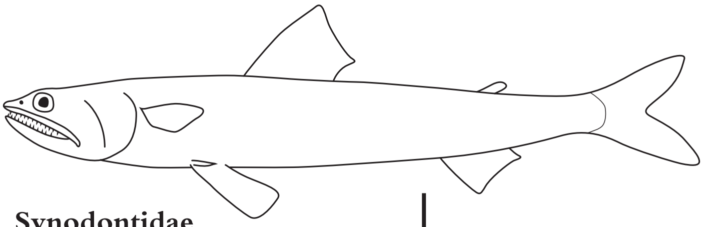

Synodontidae

B
from 6C

Pelvic fins absent (also absent in adults of *Parastromateus* in Carangidae) or if present, reduced to a short bony rudiment or with a single enlarged spine — **A** to 10A

Pelvic fins present, not reduced to a bony rudiment — **B** to 11A

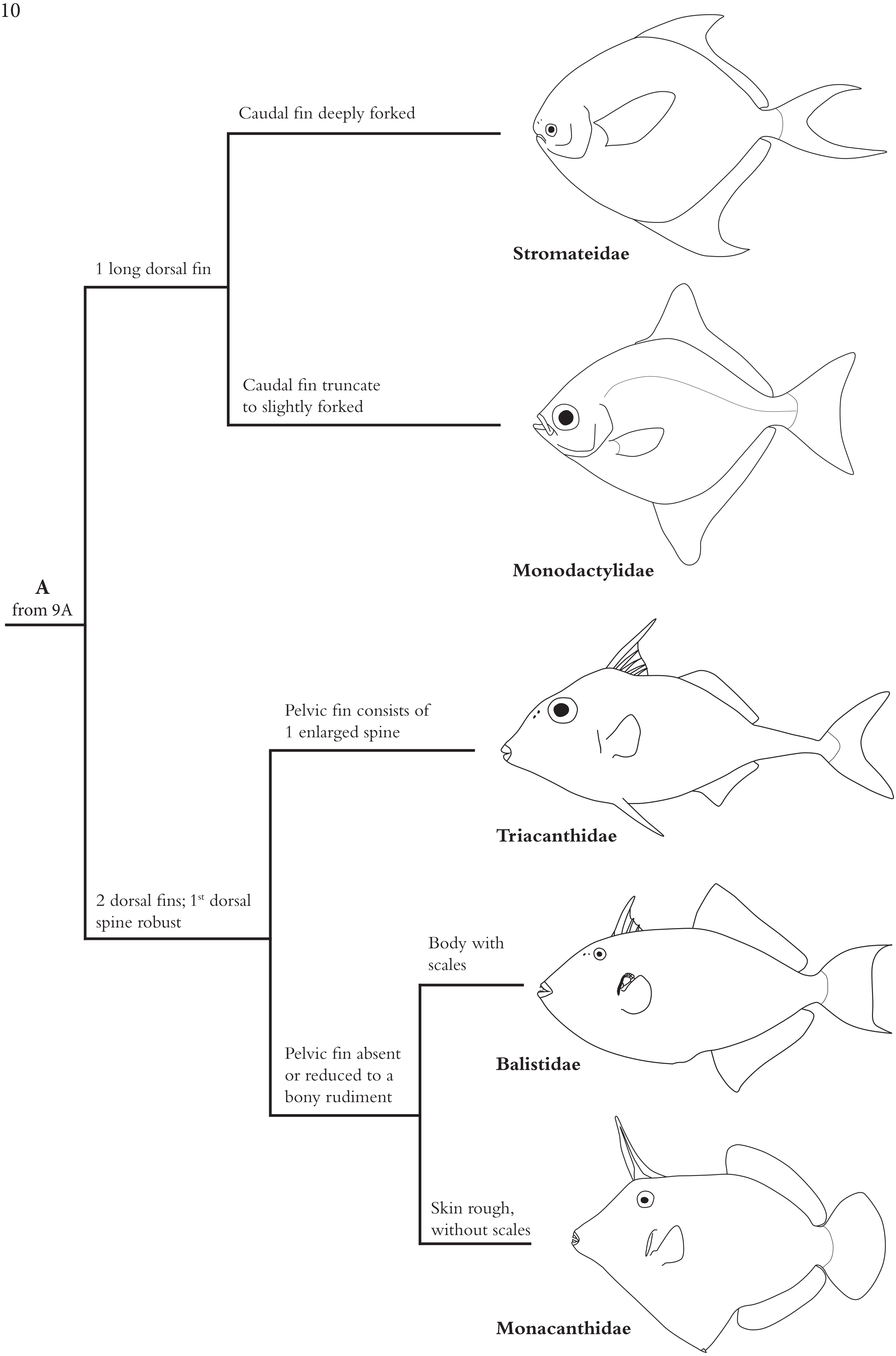
Caudal fin deeply forked
Stromateidae
1 long dorsal fin
Caudal fin truncate
to slightly forked
Monodactylidae
A
from 9A
Pelvic fin consists of
1 enlarged spine
Triacanthidae
2 dorsal fins; 1st dorsal
spine robust
Body with
scales
Balistidae
Pelvic fin absent
or reduced to a
bony rudiment
Skin rough,
without scales
Monacanthidae

A
from 9B

Pectoral-fin not leg-like; no 'fishing pole' (illicium) present on head

Pectoral-fin lobe elongate and leg-like; 1st dorsal spine modified into a 'fishing pole' (illicium)

Antennariidae

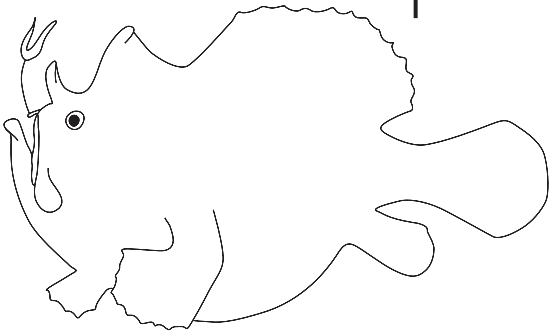

Snout not trumpet-like

Snout long and trumpet-like with a small barbel on chin; body elongate and depressed

Aulostomidae

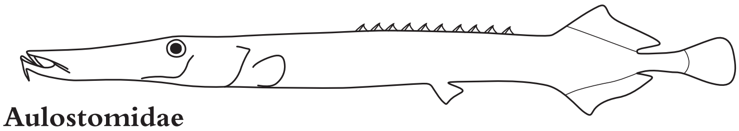

Head not bony with rostral projections and lower 3 pectoral-fin rays not free

Head large, bony with rostral projections and 3 lower rays of pectoral fins free

Triglidae

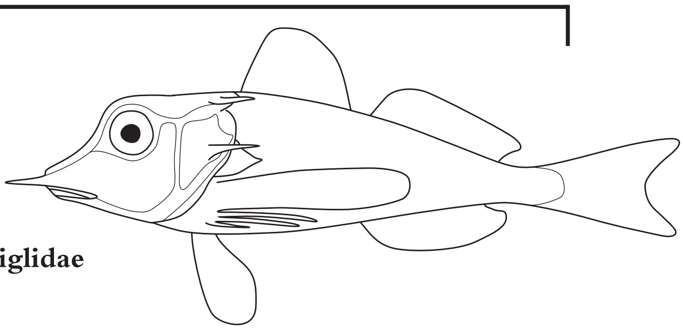

Head not strongly depressed

Head strongly depressed, with spines and bony ridges

Platycephalidae

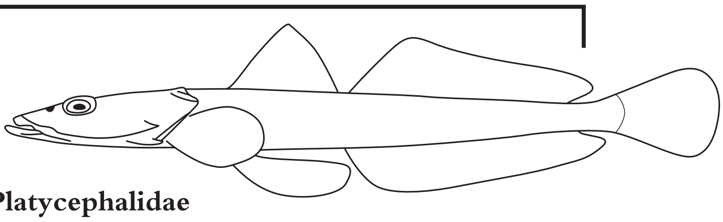

Body not oval with bright red fins

Body oval with bright red fins; usually very large

Lamprididae

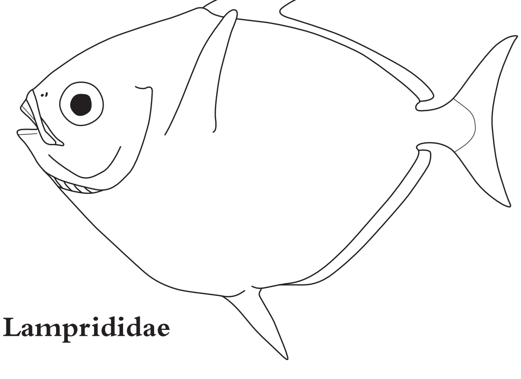

A
to 12A

A
from 11A

Usually no strong preopercular spine (if present then head not depressed; usually scales on body

Head broad and depressed, with a stout preopercular spine; no scales on body

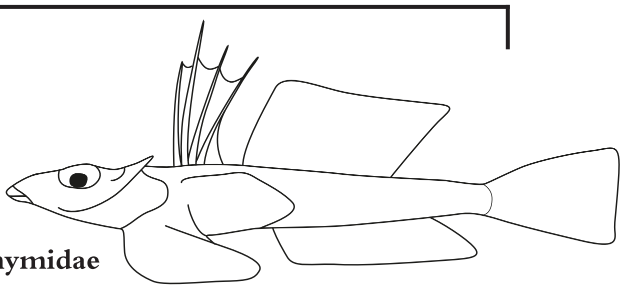

Callionymidae

Dorsal and anal fins not both sail-like, or if so then bony plates present on caudal peduncle

1st dorsal and anal fins long, high and sail-like; no bony plates on caudal peduncle

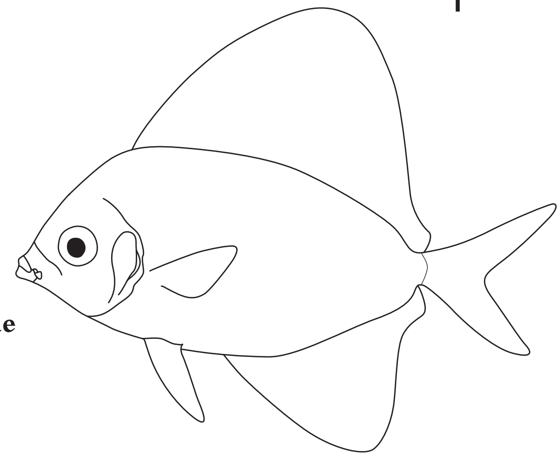

Veliferidae

No ventral keel of large scutes on belly; no mucous canals on head

A ventral keel of large scutes on belly; head with numerous mucous canals

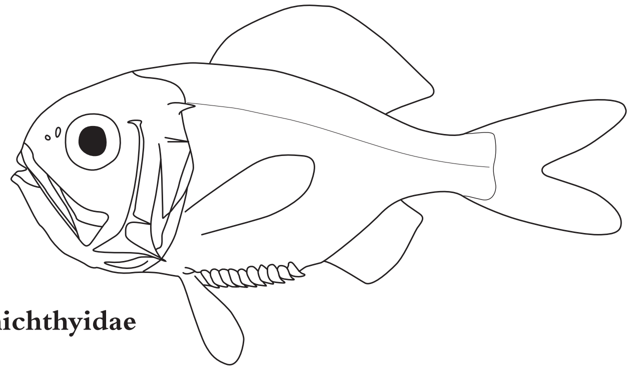

Trachichthyidae

Two dorsal fins separated by a distinct space — **A** to 13A

Only 1 dorsal fin or 2 fins separated by only a very narrow space

Head with a strong bony ridge below eye extending across cheek to preopercle; head usually with numerous spines — **B** to 14A

Head without a strong bony ridge below eye; usually none or few spines on head — **C** to 15A

A
from 12A

A pair of long barbels not present on chin

A pair of long barbels at tip of chin

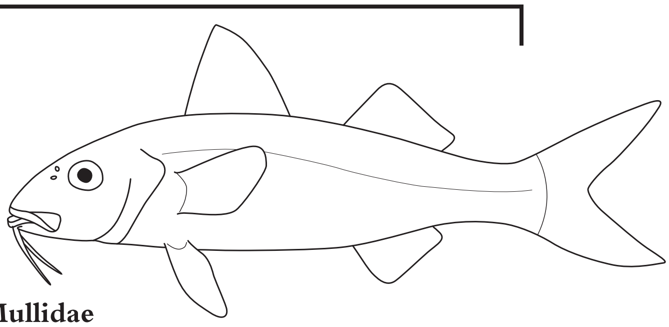

Mullidae

Lower pectoral-fin rays not filamentous

Lower pectoral-fin rays filamentous and separate from fin

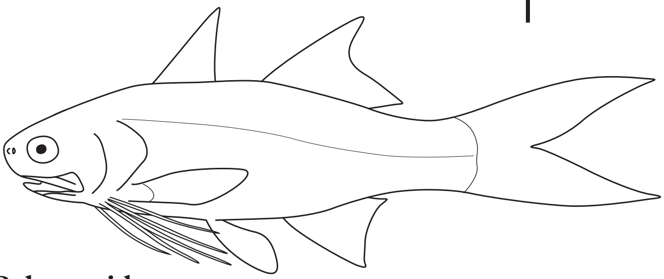

Polynemidae

Mouth not large; teeth not large

Mouth very large with many large, sharp teeth

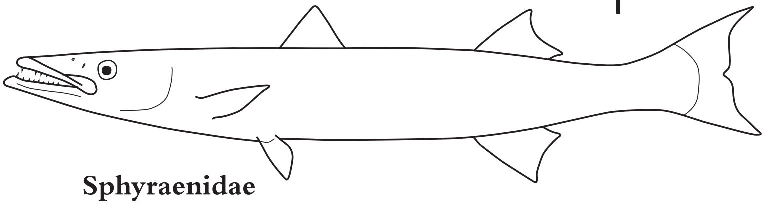

Sphyraenidae

Head broad and flattened above; no silvery band running along lateral midline of body

Head narrow, not flattened; a distinct silvery band running along lateral midline of body

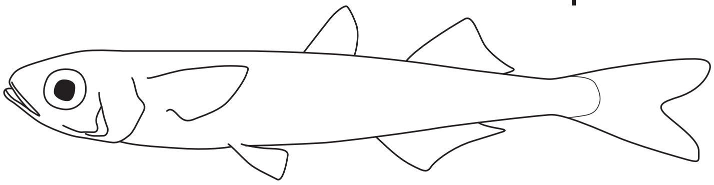

Atherinidae

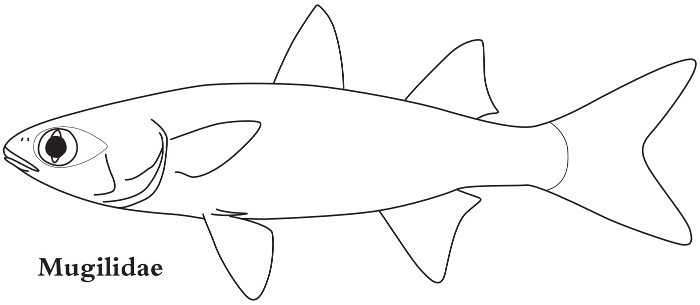

Mugilidae

A
from 12B

Dorsal-fin spines not greatly elongate; body usually without vertical reddish bars

Dorsal fin spines greatly elongate; body usually with vertical, mostly reddish, bars

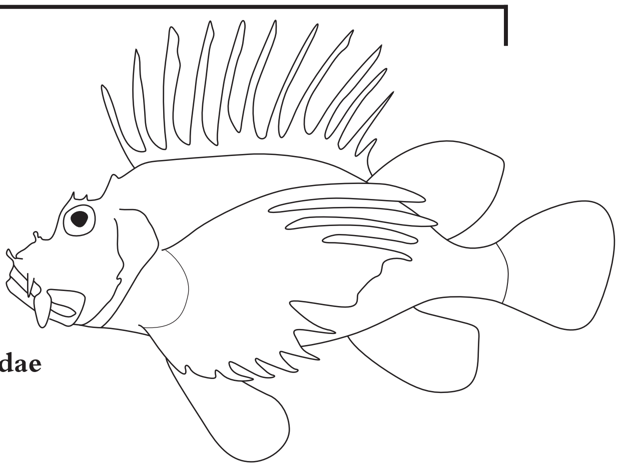

Pteroidae

Skin at gill openings connected to each other or connected to isthmus only narrowly

Skin at gill openings broadly connected to isthmus

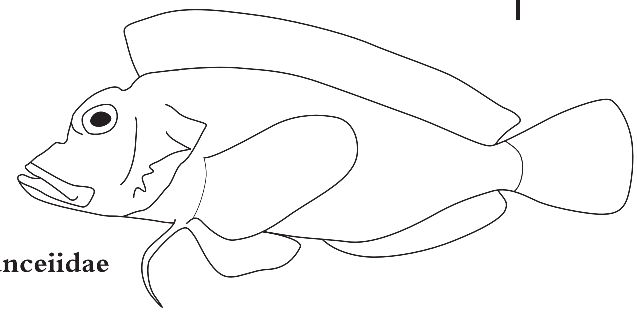

Synanceiidae

3rd infraorbital bone not inclined ventrally

3rd infraorbital bone inclined ventrally

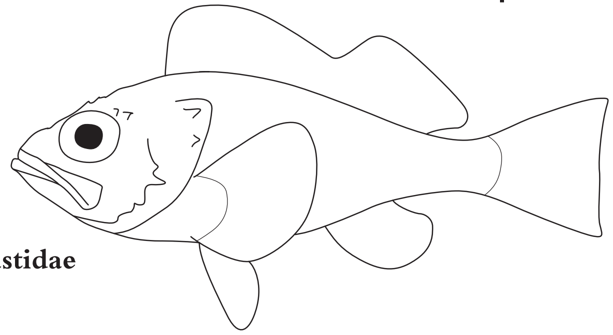

Sebastidae

Longest dorsal spines shorter than body depth

Longest dorsal spines equal to body depth

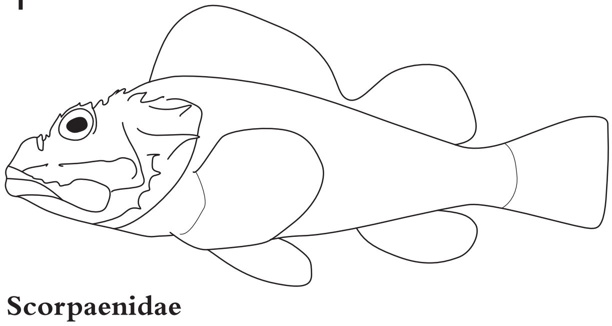

Scorpaenidae

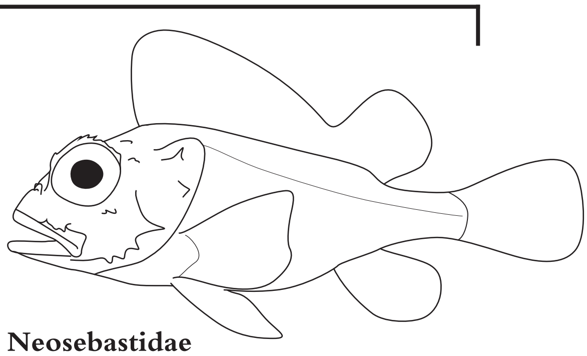

Neosebastidae

A
from 12C

A pair of long barbels not present on chin

Eyes on top of head; cleithral spine present on upper opercle

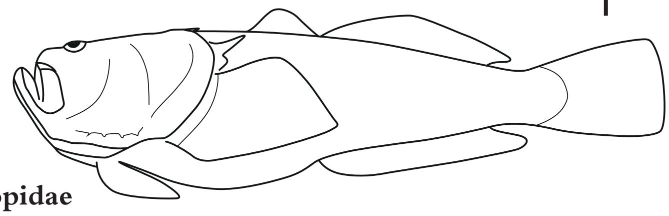

Uranoscopidae

Caudal peduncle without enlarged bony plates or spines

Caudal peduncle with 1-10 bony plates or a folding forward pointing spine on each side

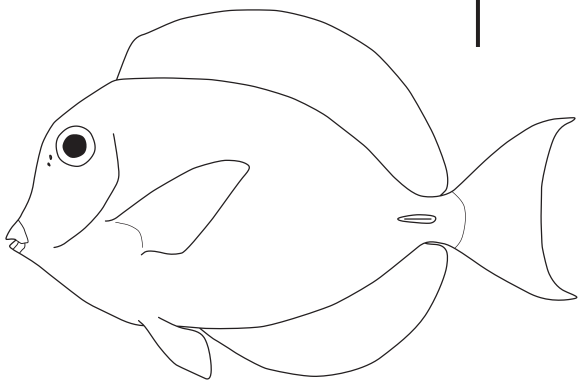

Acanthuridae

1 long dorsal fin, greatly elevated anteriorly

Snout protruding

Scales very small

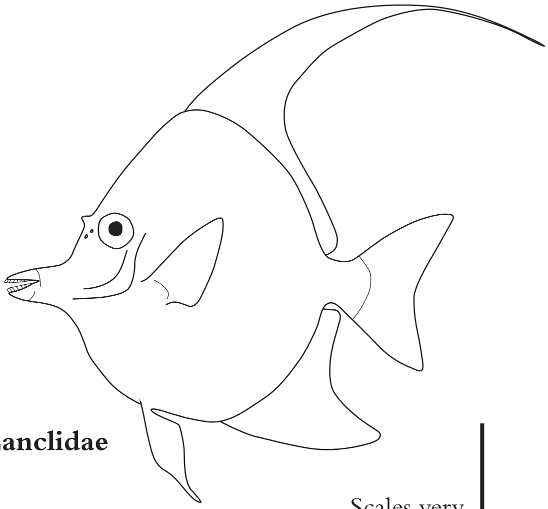

Zanclidae

Scales moderately large

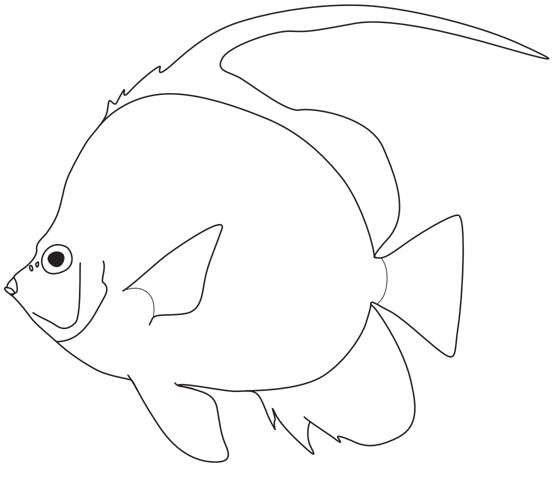

Chaetodontidae
genus *Heniochus*

Snout not protruding

A
to 16A

2 dorsal fins or a single long dorsal fin not greatly elevated anteriorly

B
to 16B

A
from 15A

Caudal fin deeply forked to very lunate; scales very large

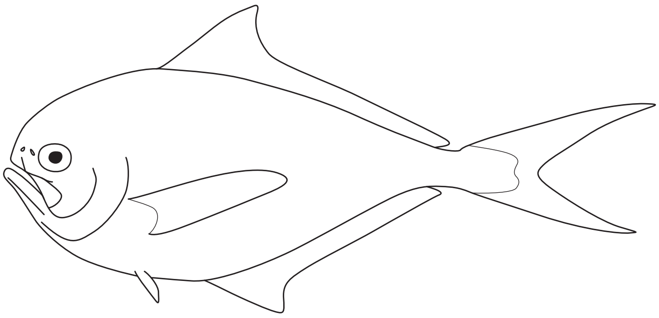

Bramidae

Caudal fin not deeply forked or lunate; scales moderately large or small

Mouth small; colour not pinkish

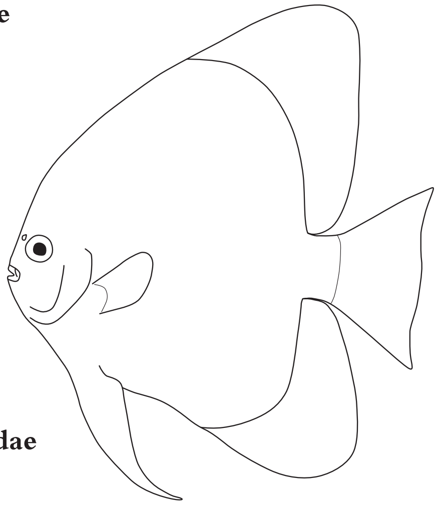

Ephippidae

Mouth large; pinkish in colour

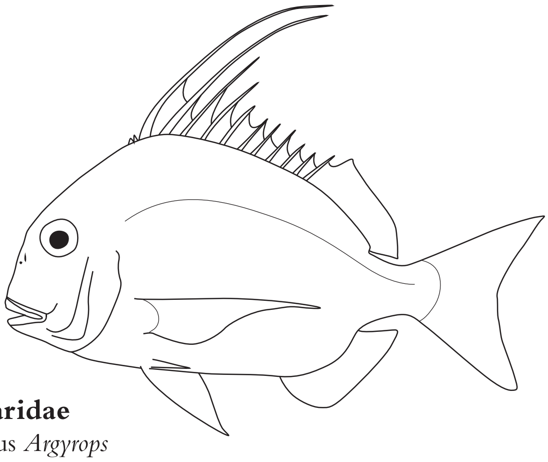

Sparidae
genus *Argyrops*

B
from 15B

Enlarged scutes present on caudal peduncle

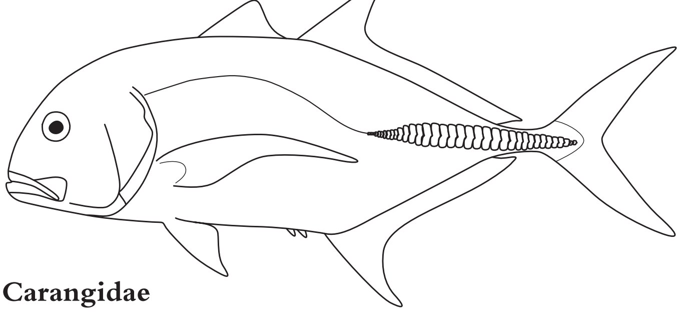

Carangidae

No enlarged scutes present on caudal peduncle

A
to 17A

A
from 16A

Pelvic fin without rays between 2 strong spines; no forward-pointing spine at front of dorsal fin

Pelvic fin with 2 strong spines with 3 soft rays inbetween; a sharp forward-poiting spine at front of dorsal fin

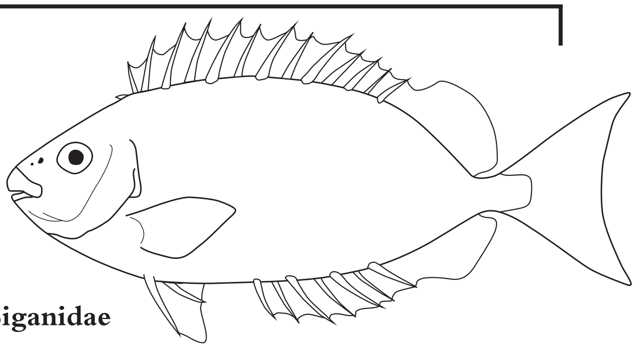

Siganidae

Body not deep, or if deep then without an angular dorsal profile and not pinkish red

Body very deep, disc-shaped, strongly compressed; dorsal profile angular; pinkish red

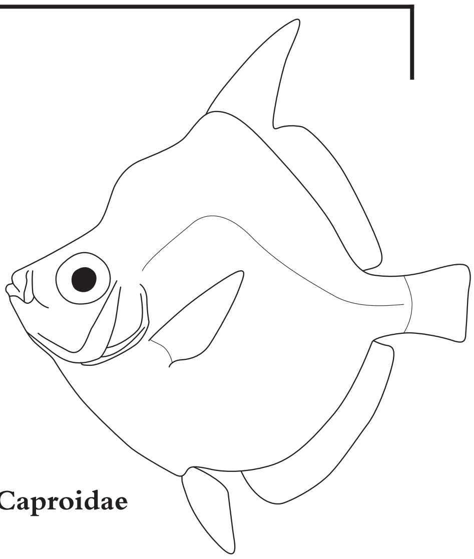

Caproidae

No cirri on nostrils or tips of dorsal spines; lower pectoral-fin rays no deeply incised

A fringe of cirri above anterior nostril and on tips of dorsal spines; lower pectoral-fin rays thickened with membranes deeply incised

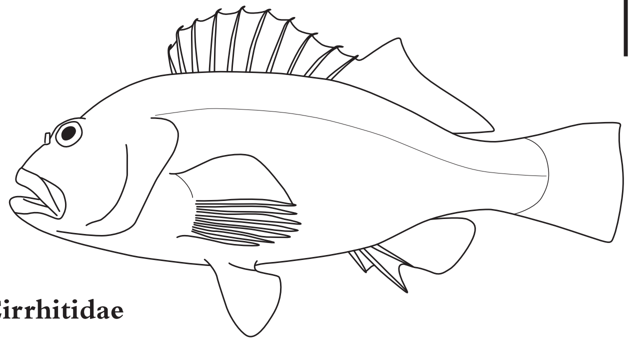

Cirrhitidae

Dorsal fin continuous, but with a deep notch between spinous and soft portions — **A** to 18A

Dorsal fin spinous portion very low, often appearing as a series of short, separate spines — **B** to 19B

Two dorsal fins clearly separate from each other by a narrow space — **C** to 20B

Dorsal fin continuous with only a shallow notch or no notch between spinous and soft portions — **D** to 21B

A
from 17A

Caudal fin slightly to deeply forked

A large and strong spine on preopercle

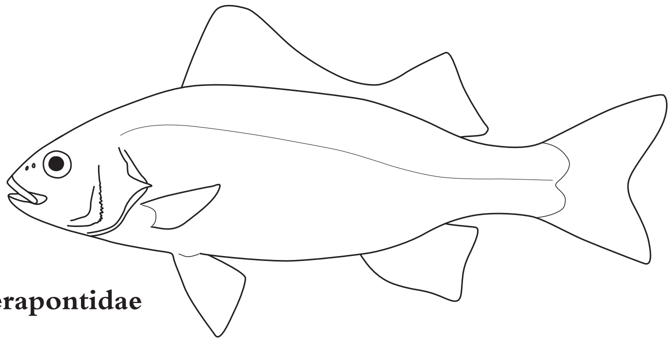

Terapontidae

No, or only short, preopercular spines

Scales strongly adherent; body mostly bright red in colour

Body relatively short; spinous dorsal fin longer than soft portion

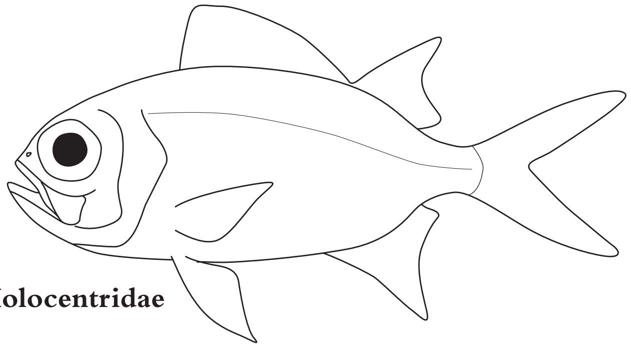

Holocentridae

Body moderately elongate; spinous and soft dorsal-fin portions subequal in length

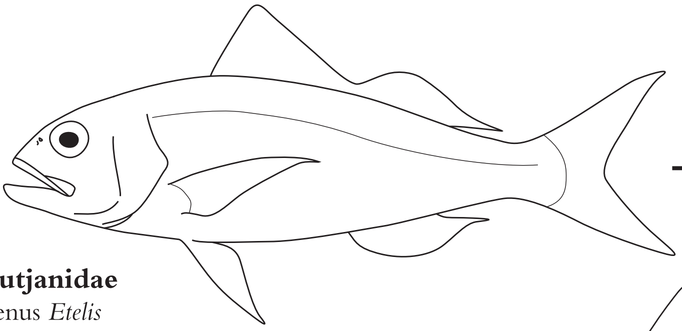

Lutjanidae
genus *Etelis*

Scales not strongly adherent, easily removed; body silvery to pinkish in colour

No enlarged flat spines on upper opercle

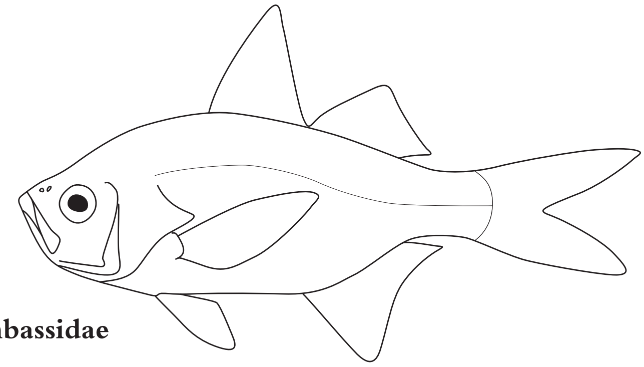

Ambassidae

2 or 3 flat spines on upper opercle

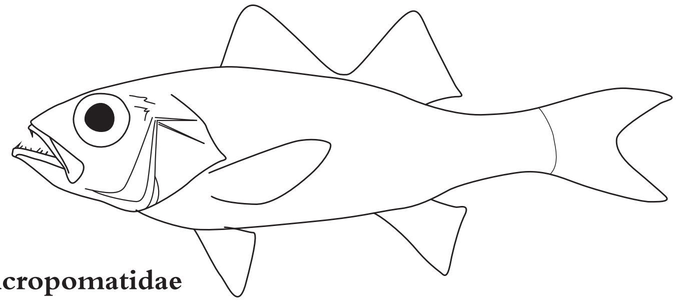

Acropomatidae

Caudal fin not forked

A
to 19A

A
from 18A

Body very deep, covered in dark spots

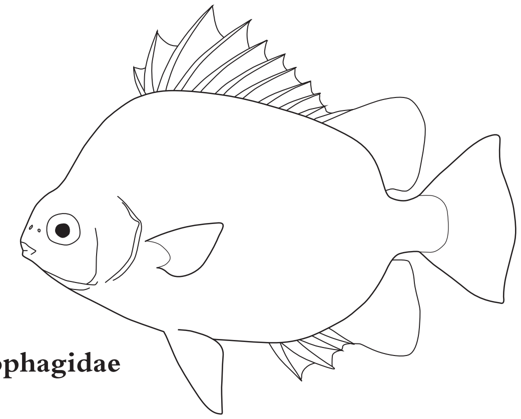

Scatophagidae

Body more elongate

Strongly patterned; body cylindrical

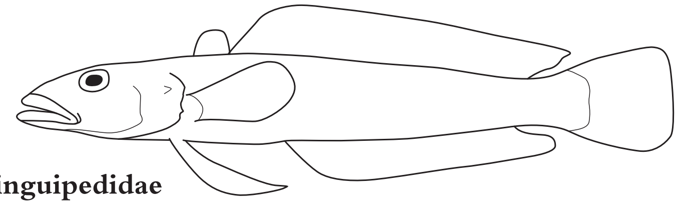

Pinguipedidae

Silvery to dusky; body moderately compressed

Head deeply concave behind eyes; preopercular margin serrated

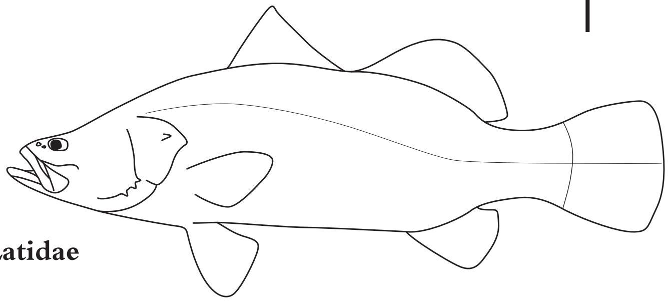

Latidae

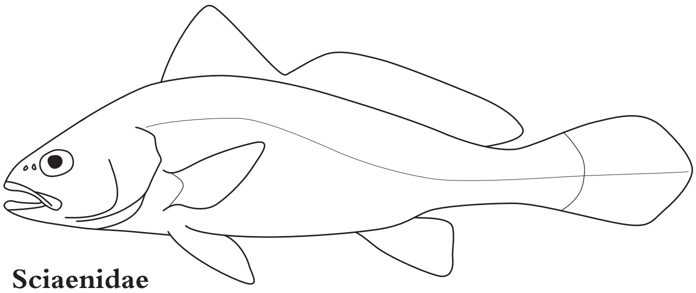

Sciaenidae

Head not deeply concave; preopercle not serrated

B
from 17B

Body elongate and cylindrical; dark brown with 2 narrow silvery stripes

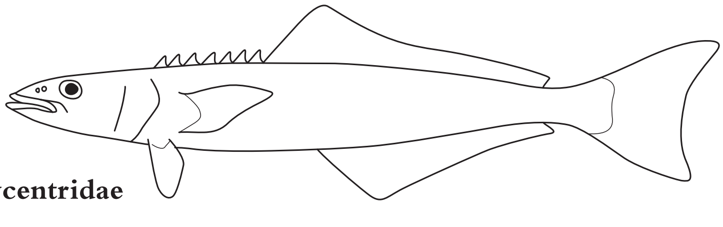

Rachycentridae

Body deeper and compressed, without stripes on sides

A
to 20A

A
from 18B

Anal fin with 2 detached spines anteriorly; body firm

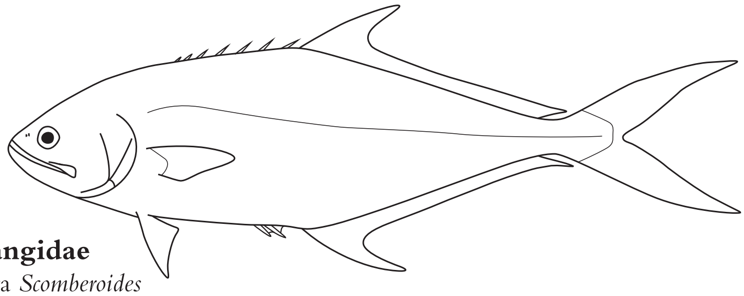

Carangidae
genera *Scomberoides* and *Trachinotus*

Anal fin without separate, detached spines anteriorly; body relatively soft

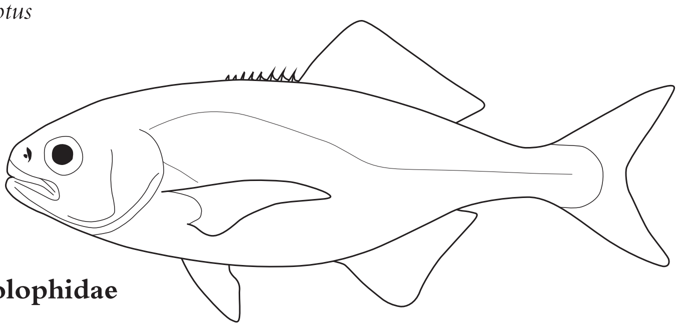

Centrolophidae

B
from 17C

Caudal fin not deeply forked

No barbel on chin; fins with spines

Small barbel present on chin, no spines in fins

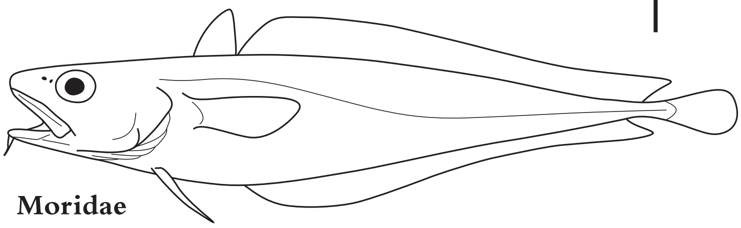

Moridae

Body with scales

Body elongate and without scales

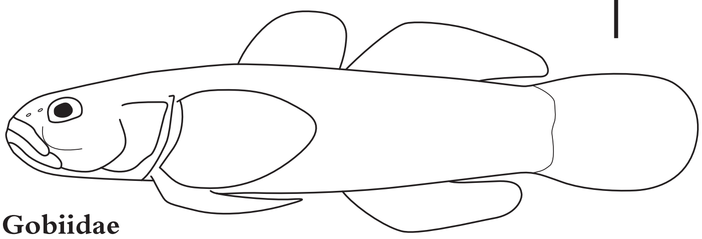

Gobiidae

Snout not long or conical; body only moderately elongate

A
to 21A

Snout long and conical; body elongate

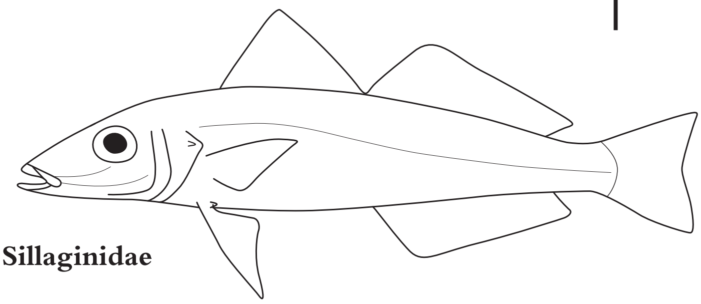

Sillaginidae

Caudal fin deeply forked

B
to 21B

A
from 20B

Preopercle with distinct double-edge; a single small opercular spine

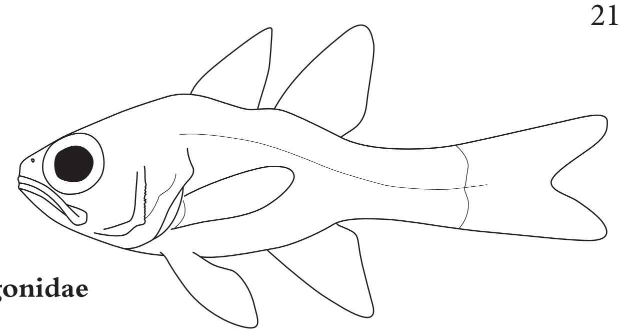

Apogonidae

Preopercle not double-edged; 2 or 3 flat opercular spines

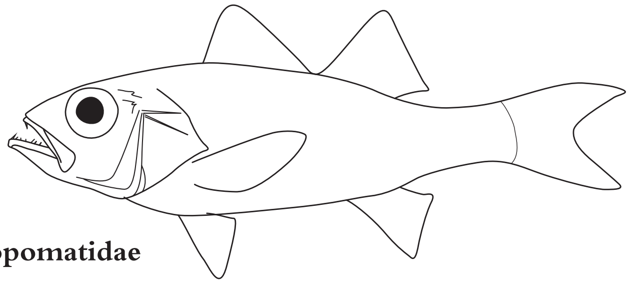

Acropomatidae

B
from 20B

No separate spines between dorsal fins

1-3 short, separate spines between dorsal fins

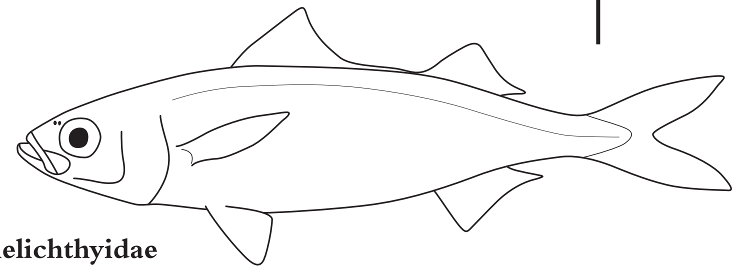

Emmelichthyidae

No detached finlets behind dorsal and anal fins

Detached finlets present behind dorsal and anal fins

No fang-like teeth at front of jaws; usually with dark spots, bars or stripes

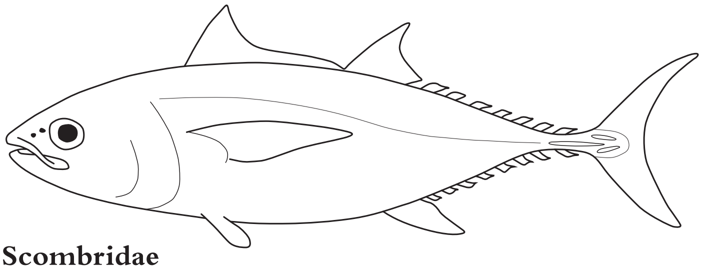

Scombridae

Fang-like teeth at front of jaws; no markings on body

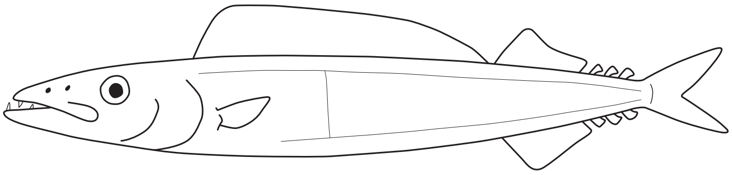

Gempylidae

A
to 22A

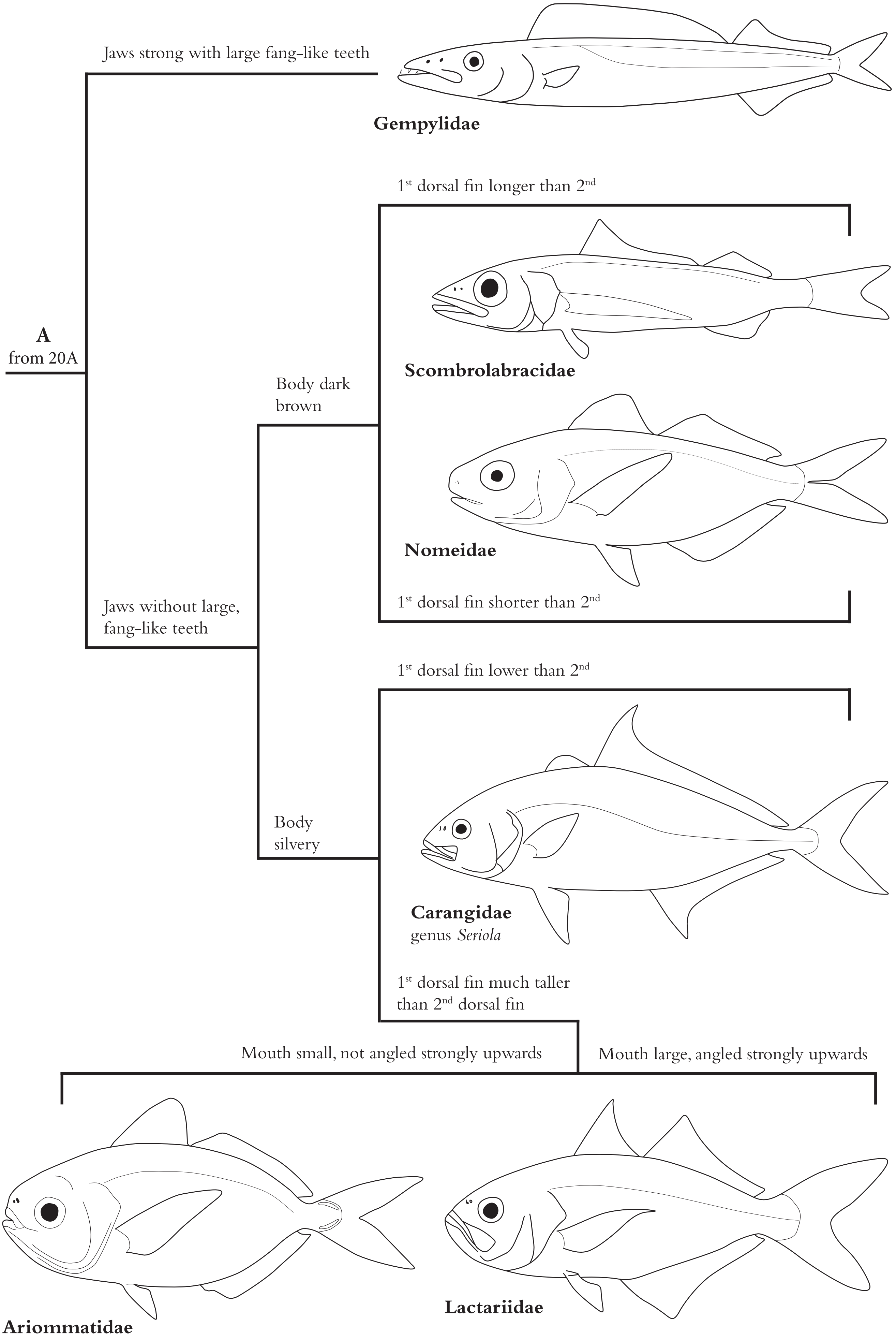
A
from 20A
Jaws strong with large fang-like teeth
Gempylidae
Jaws without large, fang-like teeth
Body dark brown
1st dorsal fin longer than 2nd
Scombrolabracidae
Nomeidae
1st dorsal fin shorter than 2nd
Body silvery
1st dorsal fin lower than 2nd
Carangidae
genus Seriola
1st dorsal fin much taller than 2nd dorsal fin
Mouth small, not angled strongly upwards
Mouth large, angled strongly upwards
Ariommatidae
Lactariidae

A
from 17D

Caudal fin deeply forked | Caudal fin not deeply forked **A** to 25B

Dorsal and anal fins not very long; head not very blunt | Dorsal and anal fins very long; body elongate; head very blunt

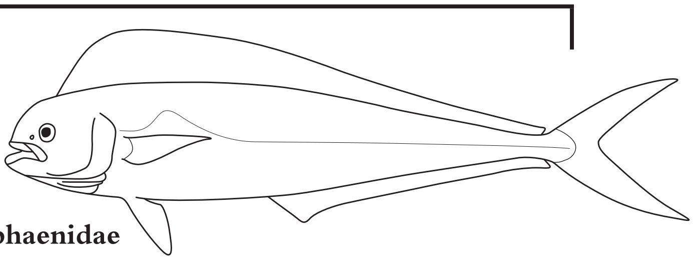

Coryphaenidae

Body not very deep and extremely compressed; anal fin not very long | Body very deep, almost triangular and extremely compressed; anal fin very long and low

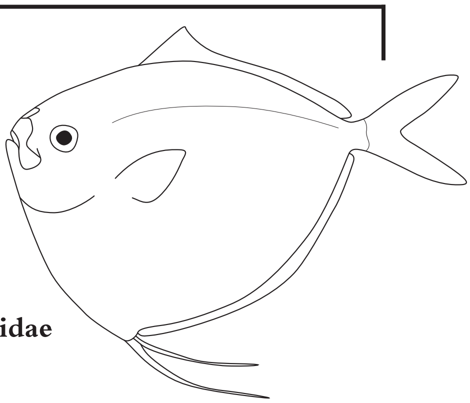

Menidae

Anal fin long, almost length of dorsal fin | Anal fin short to moderate, shorter than dorsal fin **B** to 24A

Body silvery; mouth protrusible; small in size

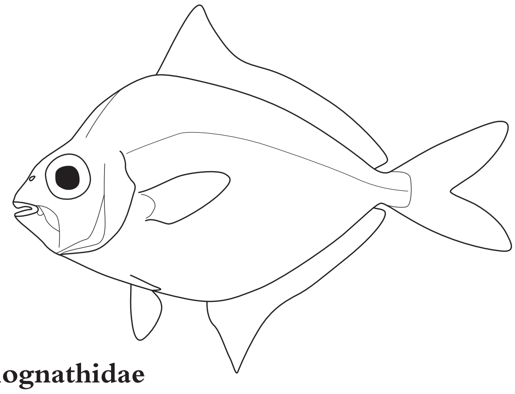

Leiognathidae

Body dark brown to dusky; mouth not protrusible; medium to large in size

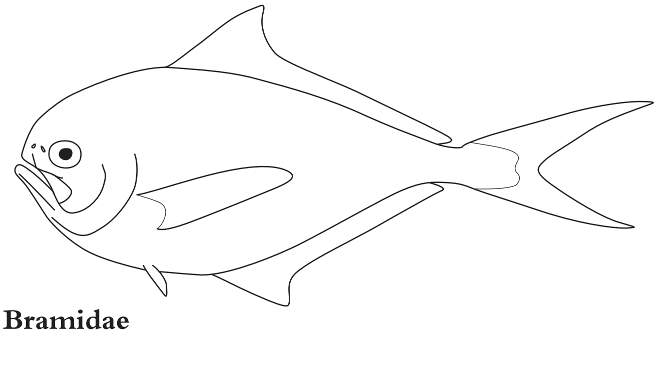

Bramidae

Scales easily removed; mouth protractile, extending downwards

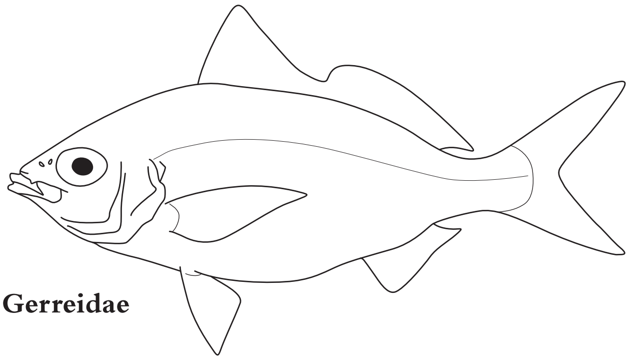

Gerreidae

A
from 23B

Anal-fin with 2 spines and 9-16 soft rays; 1 nostril on each side of head

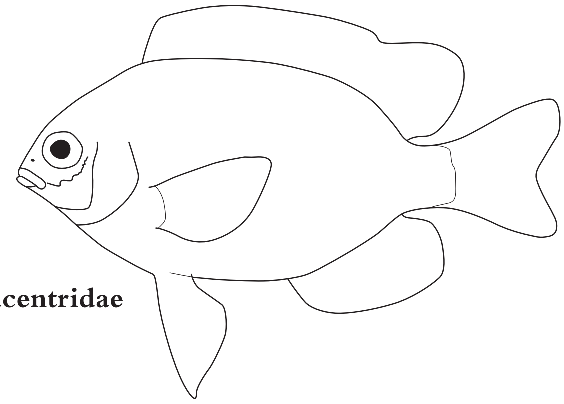

Scales adherant; mouth not protracting downwards

Pomacentridae

Anal-fin with 3 spines and 7-13 soft rays; 2 nostrils on each side of head

usually 10 (up to 15) dorsal-fin spines

9 dorsal-fin spines

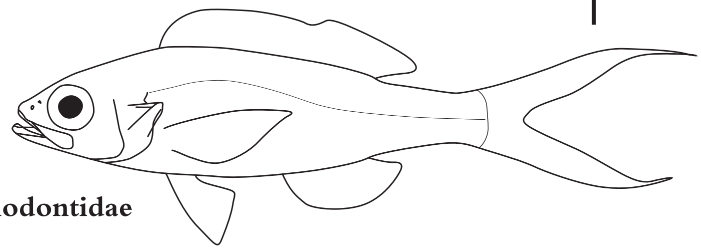

Symphysanodontidae

mostly 10-15 dorsal-fin soft rays; 8-13 anal-fin soft rays

9 dorsal-fin soft rays; usually 7 anal-fin soft rays

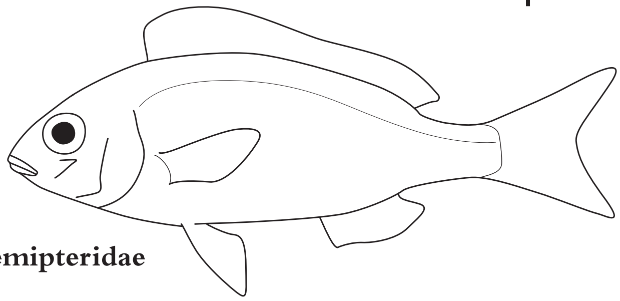

Nemipteridae

A
to 25A

A
from 24A

Mouth relatively large

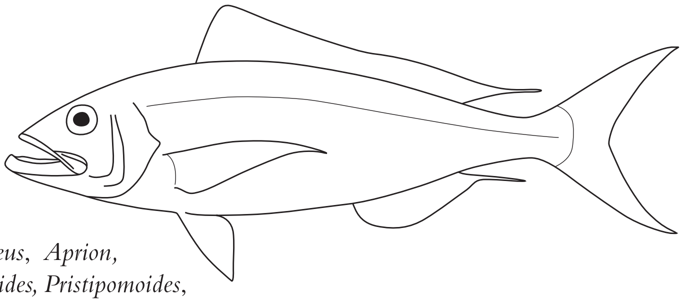

Lutjanidae
genera *Aphareus*, *Aprion*, *Parapristipomoides*, *Pristipomoides*, some *Paracaesio*

Mouth small

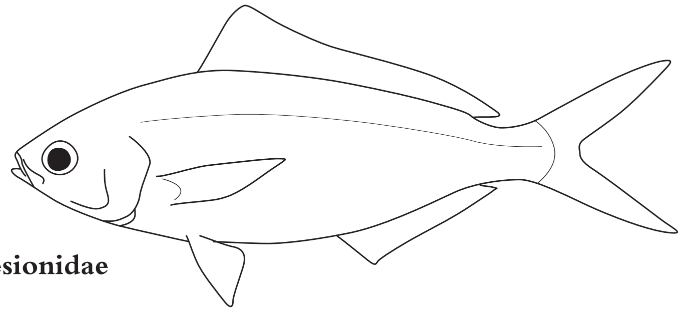

Caesionidae

B
from 23A

Caudal fin lunate

Scales large; body slimy to touch

Teeth in jaws fused to form a parrot-like beak (except in *Calotomus*); lips continuous with facial skin

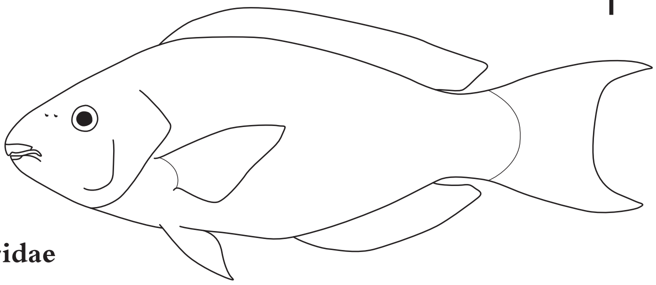

Scaridae

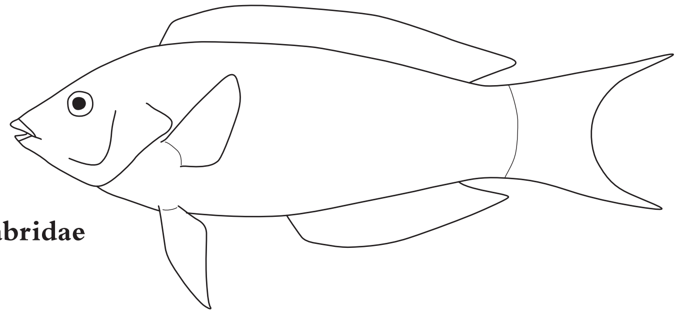

Labridae

Teeth in jaws separate, not forming a parrot-like beak; lips well-developed and not continuous with facial skin

Scales smaller; body not slimy — **A** to 26A

Caudal fin not lunate — **B** to 26B

A
from 25A

Eyes very large; mouth upturned

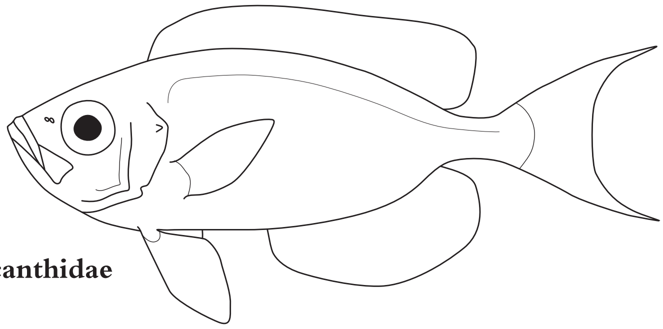

Priacanthidae

Eyes not very large; mouth not upturned

12–17 dorsal-fin soft rays

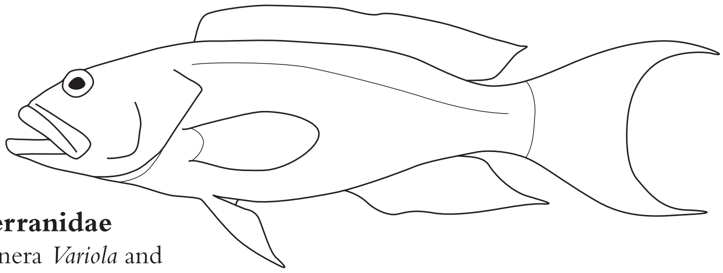

Serranidae
genera *Variola* and some anthiines

9 dorsal-fin soft rays

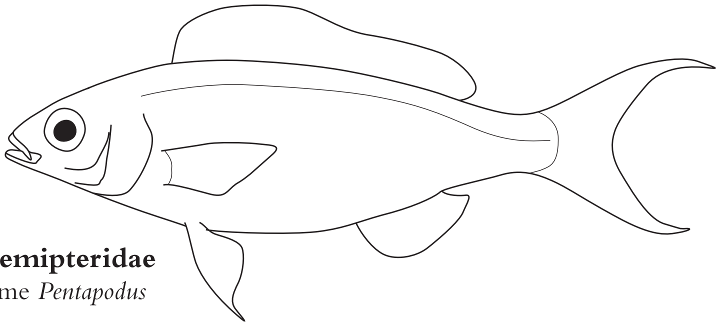

Nemipteridae
some *Pentapodus*

B
from 25B

Head not bulbous with very large eyes

Head bulbous with very large eyes;

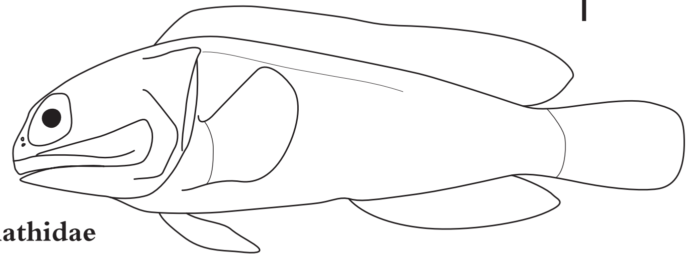

Opistognathidae

Body with scales

A
to 27A

Body without scales and elongate

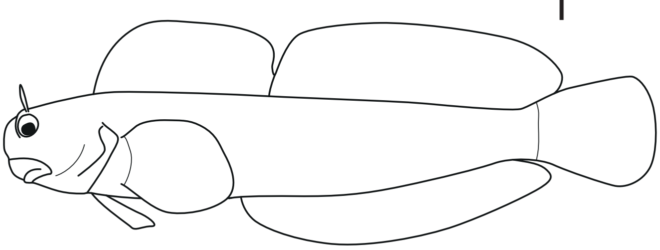

Blenniidae

A
from 26B

Caudal fin rounded

Caudal fin not rounded

A
to 29B

Soft portions of anal and dorsal fins not extending beyond base of caudal fin

Soft portions of anal and dorsal fins broadly rounded and extending beyond base of caudal fin

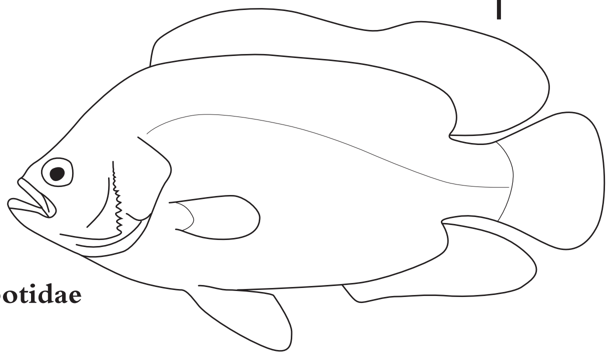

Lobotidae

Body not very deep; usually not silvery, if so then also with bright colours

Body very deep; mostly silvery in colour

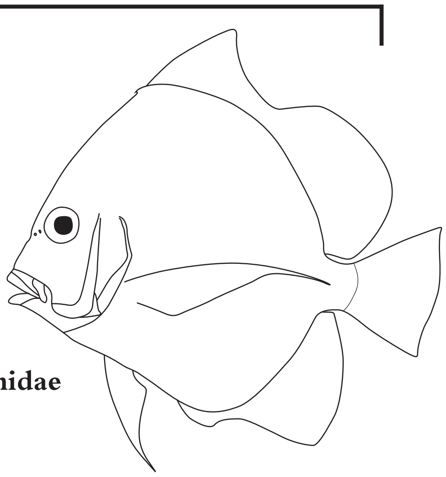

Drepaenidae

Scales not very large, tough and slimy

Scales very large, tough and slimy to touch

B
to 28A

1-3 dorsal-fin spines

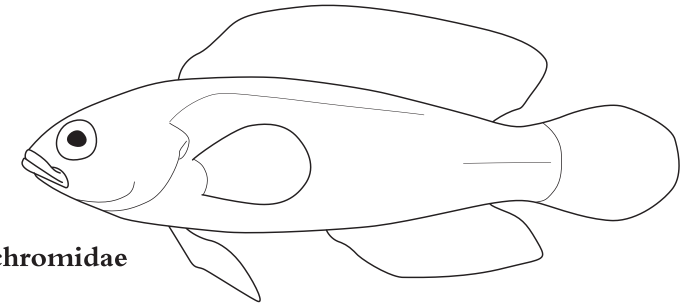

Pseudochromidae

6-16 dorsal-fin spines

C
to 28B

A
from 27B

Teeth in jaws fused to form a parrot-like beak (except in *Calotomus*); lips continuous with facial skin

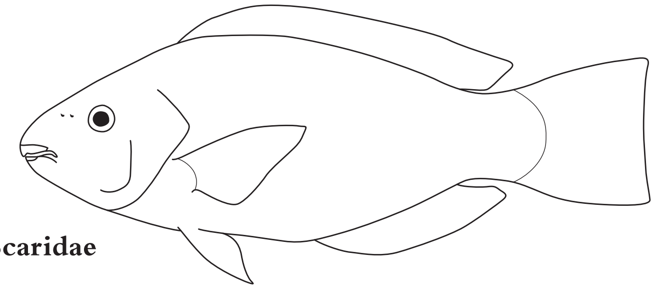

Scaridae

Teeth in jaws separate, not forming a parrot-like beak; lips well-developed and not continuous with facial skin

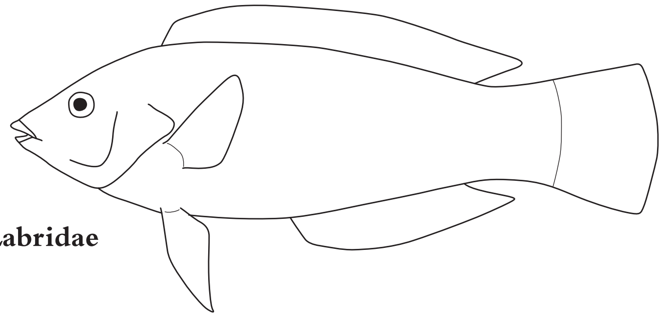

Labridae

B
from 27C

2nd anal-fin spine not robust; no flap on nostrils

2nd anal-fin spine long and robust; large flap on anterior nostril

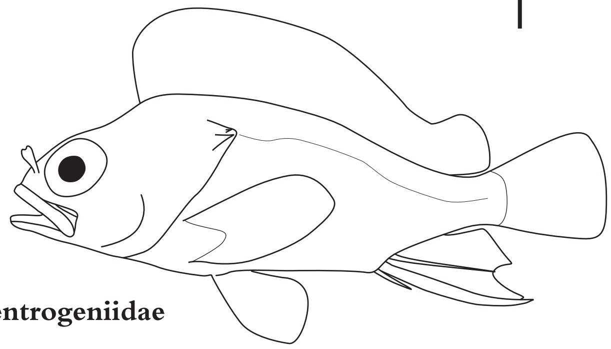

Centrogeniidae

Body deep and strongly compressed; no spines on upper opercle

Body mostly moderately elongate and only moderately compressed; 3 spines on upper opercle

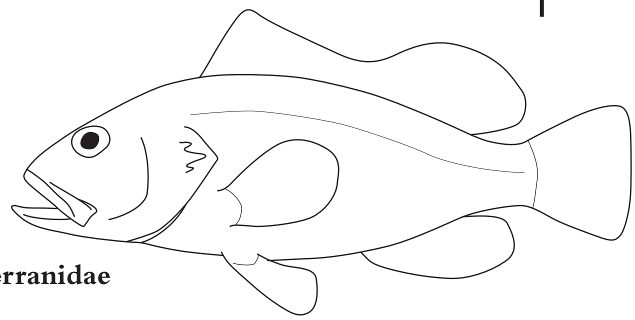

Serranidae

A
from 29A

A
from 28B

1 or more strong spines at angle of preopercle

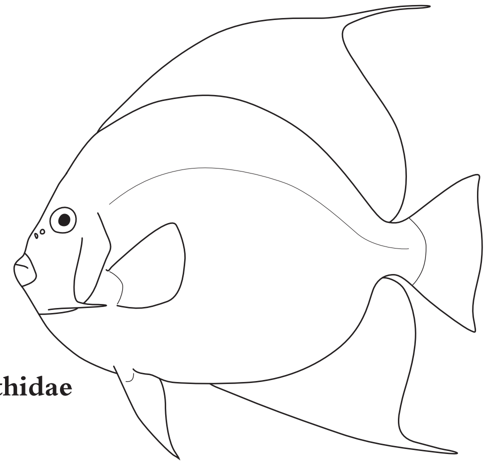

Pomacanthidae

No spines at angle of preopercle

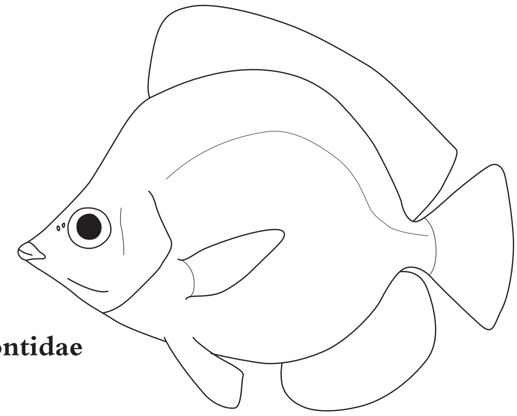

Chaetodontidae

B
from 27A

Dorsal fin not far back on body; lower jaw not distinctly protruding; dorsal head profile not flat

Dorsal fin situated far back on body; lower jaw protruding; dorsal head profile flat

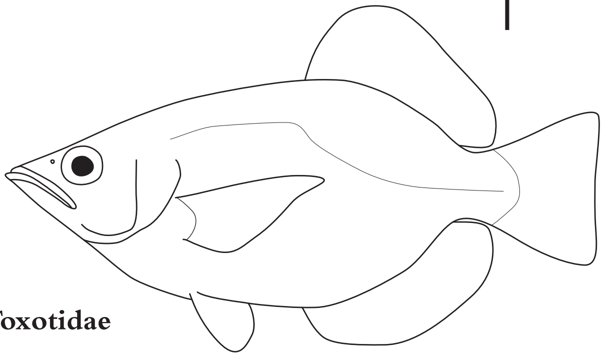

Toxotidae

Body not deeper than long, usually not strongly compressed, if so then brightly coloured

Body deeper than long and strongly compressed; silvery to blackish in colour

A
to 30A

Scales very large, tough and slimy to touch

B
to 30B

Scales not very large, tough and slimy

C
to 30C

A
from 29A

Pectoral fins not greatly elongate

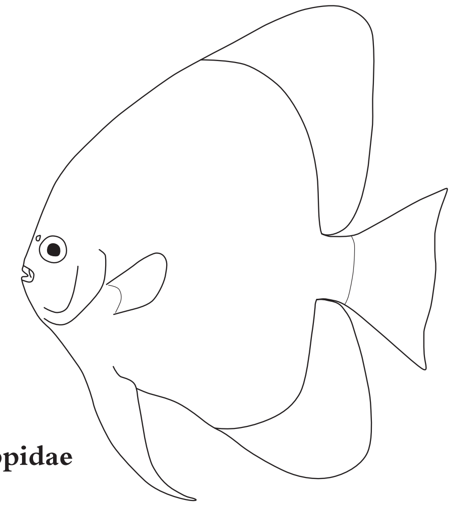

Ephippidae

Pectoral fins greatly elongate

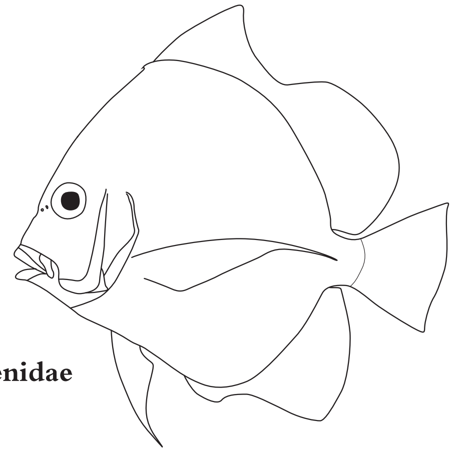

Drepaenidae

B
from 29B

Teeth in jaws fused to form a parrot-like beak (except in *Calotomus*); lips continuous with facial skin

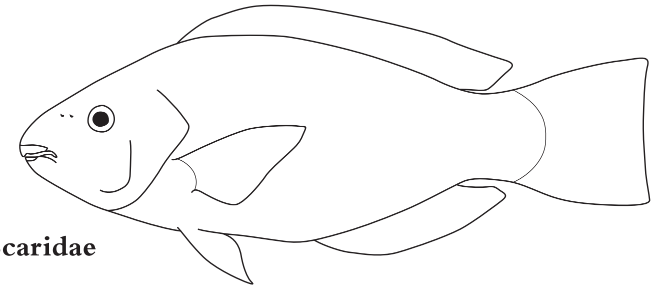

Scaridae

Teeth in jaws separate, not forming a parrot-like beak; lips well-developed and not continuous with facial skin

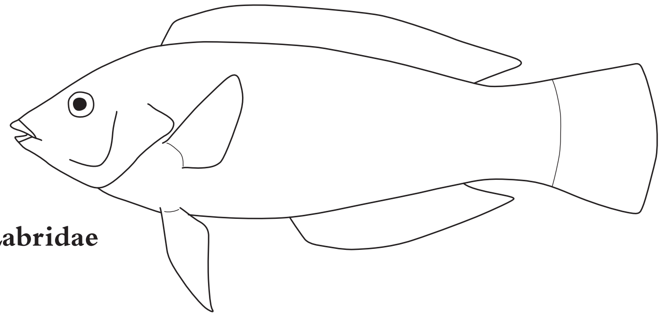

Labridae

A
from 29C

Eyes not very large; pelvic fins behind pectoral fins; mouth not angled upwards

Eyes very large; pelvic fins in front of pectoral fins; mouth angled upwards

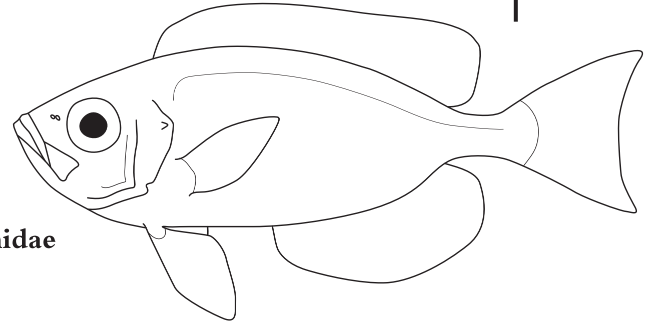

Priacanthidae

1 or 2 anal-fin spines

Body relatively elongate; 1 soft spine on upper opercle

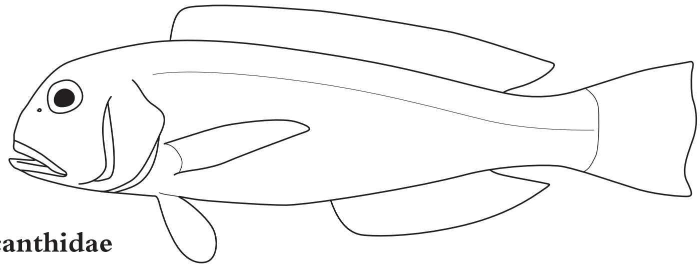

Malacanthidae

Body moderately deep; no spine on upper opercle

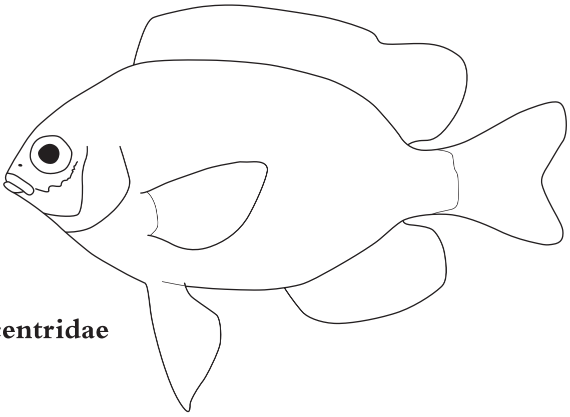

Pomacentridae

3 or more anal-fin spines

3 spines on upper opercle

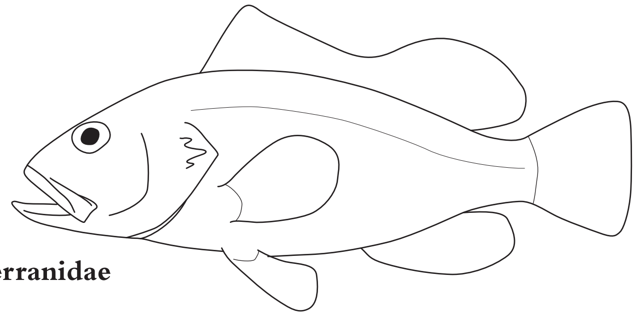

Serranidae

0–2 (usually 0) spines on upper opercle

A
to 32A

A
from 31A

Body moderately elongate; mouth larger, not protrusible

Body very deep; mouth small and protrusible

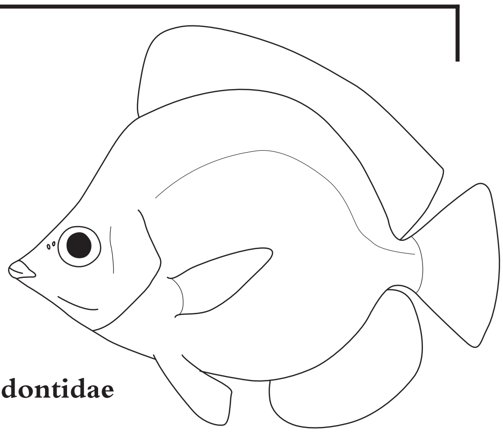

Chaetodontidae

Chin without pores; 2nd anal-fin spine not very strong

Chin with 2 or 6 pores; 2nd anal-fin spine usually very strong

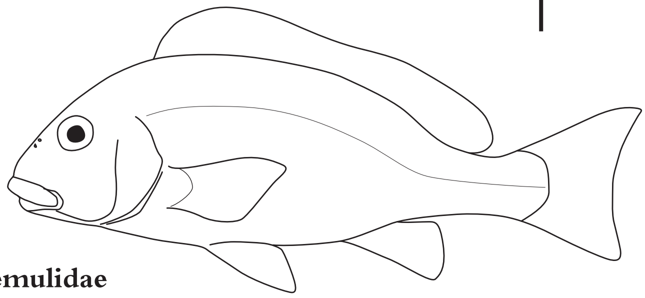

Haemulidae

No strong spine on posterior edge of opercle

Opercle with 2 spines on posterior edge, lower one strong

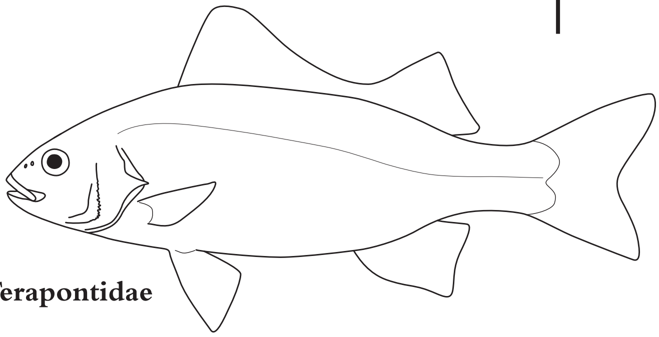

Terapontidae

Teeth usually in more than one row and not incisor-like

Teeth in both jaws in a single row and incisor-like

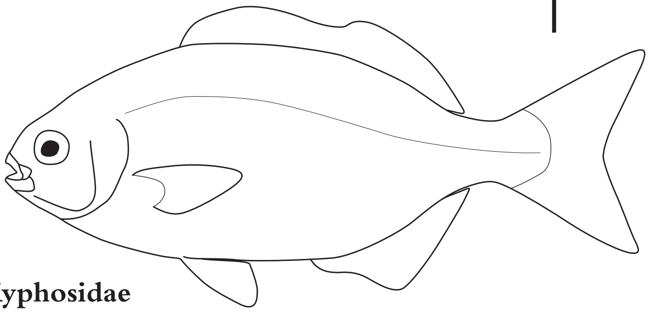

Kyphosidae

A
to 33A

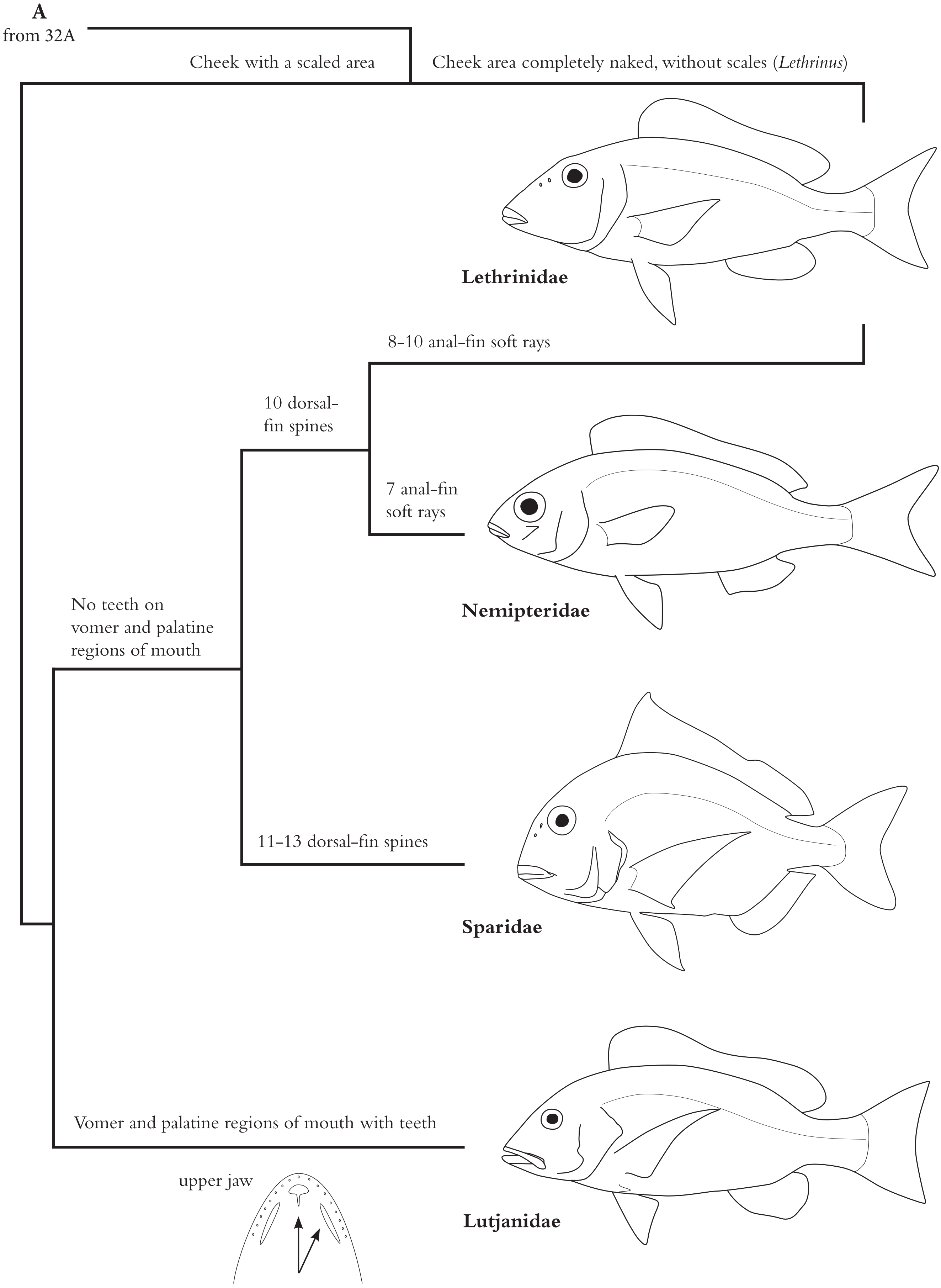
A
from 32A
Cheek with a scaled area
Cheek area completely naked, without scales (*Lethrinus*)
Lethrinidae
8-10 anal-fin soft rays
10 dorsal-fin spines
7 anal-fin soft rays
Nemipteridae
No teeth on vomer and palatine regions of mouth
11-13 dorsal-fin spines
Sparidae
Vomer and palatine regions of mouth with teeth
upper jaw
Lutjanidae
